# Characterization of a novel peptide mined from the Red Sea brine pools and modified to enhance its anticancer activity

**DOI:** 10.1186/s12885-023-11045-4

**Published:** 2023-07-26

**Authors:** Youssef T. Abdou, Sheri M. Saleeb, Khaled M. A. Abdel-Raouf, Mohamed Allam, Mustafa Adel, Asma Amleh

**Affiliations:** 1 Biotechnology Program, American University in Cairo, New Cairo, Egypt; 2 Biology Department, American University in Cairo, New Cairo, Egypt

**Keywords:** Anticancer peptides, Antimicrobial peptides, AUC Red Sea metagenomics database, Cancer cell lines, Hepatocellular carcinoma, Ovarian cancer cells

## Abstract

Drug resistance is a major cause of the inefficacy of conventional cancer therapies, and often accompanied by severe side effects. Thus, there is an urgent need to develop novel drugs with low cytotoxicity, high selectivity and minimal acquired chemical resistance. Peptide-based drugs (less than 0.5 kDa) have emerged as a potential approach to address these issues due to their high specificity and potent anticancer activity. In this study, we developed a support vector machine model (SVM) to detect the potential anticancer properties of novel peptides by scanning the American University in Cairo (AUC) Red Sea metagenomics library. We identified a novel 37-mer antimicrobial peptide through SVM pipeline analysis and characterized its anticancer potential through in silico cross-examination. The peptide sequence was further modified to enhance its anticancer activity, analyzed for gene ontology, and subsequently synthesized. To evaluate the anticancer properties of the modified 37-mer peptide, we assessed its effect on the viability and morphology of SNU449, HepG2, SKOV3, and HeLa cells, using an MTT assay. Additionally, we evaluated the migration capabilities of SNU449 and SKOV3 cells using a scratch-wound healing assay. The targeted selectivity of the modified peptide was examined by evaluating its hemolytic activity on human erythrocytes. Treatment with the peptide significantly reduced cell viability and had a critical impact on the morphology of hepatocellular carcinoma (SNU449 and HepG2), and ovarian cancer (SKOV3) cells, with a marginal effect on cervical cancer cell lines (HeLa). The viability of a human fibroblast cell line (1Br-hTERT) was also significantly reduced by peptide treatment, as were the proliferation and migration abilities of SNU449 and SKOV3 cells. The annexin V assay revealed programmed cell death (apoptosis) as one of the potential cellular death pathways in SNU449 cells upon peptide treatment. Finally, the peptide exhibited antimicrobial effects on both gram-positive and gram-negative bacterial strains. The findings presented here suggest the potential of our novel peptide as a potent anticancer and antimicrobial agent.

**Supplementary Information:**

The online version contains supplementary material available at 10.1186/s12885-023-11045-4.

## Introduction

Peptide-based drugs have attracted attention in recent years for their potential application in anticancer and antimicrobial therapies. They offer greater specificity, fewer off-site side effects, and increased potency compared with current cancer therapies [1]. Protein-based biologics are complex peptide molecules with a typical molecular weight exceeding 5 KDa; synthesizing these molecules is expensive and challenging [2]. On the other hand, conventional small-molecule drugs with molecular weights less than 0.5 KDa, tend to exhibit lower specificity and often induce off-target side effects [3]. Peptide drugs fall within the molecular weight range of 0.5 to 5 KDa, offering a unique combination of biologics' specificity, potency, and low toxicity, along with the advantages of easy production and metabolic stability of small-molecule drugs [2]. Anticancer peptides (ACPs) comprise one class of peptide drugs and are relatively small molecules (5 to 50 amino acids) with cationic and amphiphilic properties [2]. Due to these properties, ACPs specifically target relatively anionic cancer cells with low reactivity with relatively neutral normal mammalian cells [4, 5]. This targeting strategy is rarely used in current cancer therapeutics. Chemotherapeutic agents do not differentiate between normal proliferating cells and cancer cells, thus cannot target indolent or dormant cancers [6]. The development of a chemoresistant phenotype further reduces the therapeutic utility of chemotherapy [7], as current cancer therapy regimens are unable to overcome multidrug resistance, which is slowly acquired via progressive tumor mass exposure [6].

The anticancer properties of ACPs are established through membranolytic (direct-acting ACPs) and/or non-membranolytic modes of action (indirect-acting, programmed cell death) [8]. In terms of membranolytic mechanisms, ACPs can disrupt the cellular, mitochondrial, or lysosomal membranes by either the carpet or barrel-stave mechanism. Small peptides can aggregate via hydrophobic interactions to form a structure through the plasma membrane resembling a traditional ion channels, or can alternatively pass through the plasma membrane and permeate the mitochondrial membrane. Here they induce swelling of the mitochondria, causing the release of cytochrome c and subsequent activation of caspases 9 and 3, or can modify the lysosomal membrane, resulting in acidification of the cytosol. The non-membranolytic mechanisms of ACP action include activation of calcium channels and consequent calcium ion influx, augmentation of proteasome activity, inhibition of pro-survival genes, or cell-cycle arrest [5, 9]. Thus, ACPs exert a non-systemic targeted effect compared with conventional chemotherapy [10]. Additionally, ACPs have been shown to promptly induce the death of cancer cells, hindering the development of resistance [11].

Some ACPs are inherently antimicrobial in nature as presented in antimicrobial peptides (AMPs) [12], and have been classified according to their secondary/tertiary structure as α-helical or β-sheets [13]. This dual property is predicted to make ACPs superior to conventional chemotherapeutic drugs by minimizing the development of cellular acquired resistance in cancer cells [10].

ACPs exhibit promising therapeutic potential against cancer, with their ability to inhibit cancer cell proliferation, migration, and angiogenesis. Moreover, ACPs possess the added advantage of being cost-effective to produce, positions them as preferable alternative to conventional chemotherapy, immunotherapy, or radiotherapy for cancer treatment.. However, the substantial cytotoxicity and poor targeting of ACPs have impaired their application. Characteristics, such as the number, charge, and sequence of the specific amino acids are altered via reconstruction mechanisms to overcome the shortfalls of the targeting effectiveness of ACPs. This reconstruction process involves modifying the amino acids that constitutes the main and/or side chains employing three distinct machine learning (ML) approaches: supervised, unsupervised, and reinforced learning.

The present study aimed to screen the American University in Cairo (AUC) Red Sea metagenomics data library, generated during AUC/KAUST Red Sea Microbiome Project, to identify a novel antimicrobial peptide with potential anticancer activity. The metagenomic samples primarily came from three locations in the Red Sea: Atlantis II Deep, Discovery Deep, and Kebrit Deep [14]. Scientists studying the chemical properties of the brine pools in Atlantis II Deep and Discovery Deep observed those of the former to be predominantly sulfur enriched, while those of the latter were carbon and nitrogen enriched. This difference in chemical composition results in unique microorganism diversity, specifically observed at the deepest soil layers in each site; specializing in metabolizing the compounds dominant in their respective sites. We identified a novel 37-residue antimicrobial peptide from the brine pool of Atlantis II Deep and modified the amino acid sequence to increase its hydrophobicity and anticancer activity. The modified 37-mer peptide was tested in a dose-dependent cytotoxicity assay on grade I, II and III/IV hepatocellular carcinoma cell lines, (HepG2 and SNU449, respectively), an ovarian cancer cell line (SKOV3), and on HeLa cervical cancer cells. The anticancer properties of the peptide were evaluated by analyzing changes in cell viability, cell morphology, and inhibition of cellular migration. Mode of cell death was also investigated, and the targeted selectivity was validated via screening of human erythrocytes. The peptide was also assessed for antimicrobial activity on both gram-positive (*Staphylococcus aureus*) and gram-negative (*Escherichia coli*) bacteria to verify its antimicrobial potential.

## Materials and methods

### Raw data and quality control

The metagenomic dataset’s raw sequences were processed and executed using PRINSEO, with the 454 GS FLX/FLX + data processing software [15, 16]. To filter out less-covered read ends, we set a threshold of 15 base pairs (bp) for the quality of the read ends. Additionally, we excluded read lengths below 60 bp and sequences with sequence entropy level below 50. An overall quality filter was applied to maintain good read depth and coverage, using a sliding window with a minimum length of 15. To eliminate exact or nearly identical 454 artificial replicates [17] with 98% sequence similarity, we utilized the CD-HIT-454 default criteria.

During the quality control process, an additional filter was implemented, requiring a mean quality score of ≥ 15, across the entire trimmed sequence length. Sequences containing ambiguity characters, > 5% of their entire length, were removed. Furthermore, we eliminated 454 exact and near identical (down to 98% similarity) artificial replicates “ghost reads sequencing artifact” [17] using CD-HIT-454 [18] default criteria.

It’s worth noting that the quality control process had no significant impact on the peptides’ length distribution or GC content distribution [15]. However, it did improve the reads annotation from public datasets by eliminating low-complexity reads, which tend to produce random alignments with low significance. Additionally, this process removed artificial duplicate reads that didn’t align with the random sampling nature associated with DNA fragmentation during sequencing library construction.

### Support Vector Machine (SVM) model

The screening process was adapted from the two anticancer peptides discovery support vector machine models described previously [12], where a set of experimentally proven anticancer peptides (ACPs) from antimicrobial databases [19–21] was compared to another set of random peptides derived from SwissProt for the first model, and a set of antimicrobial peptides (AMPs) with no anticancer activity for the second model. This method inherited their reported sensitivity, specificity, and accuracy. Classification based on amino acid composition was reported to have 88.89% sensitivity, 85.29% specificity, and 85.62% accuracy in the first model; and 73.78% sensitivity, 76.02% specificity, and 75.70% accuracy in the second model. Additionally, the classification based on dipeptide composition was reported to have 90.22% sensitivity, 84.80% specificity, and 85.29% accuracy in the first model, and 77.78% sensitivity, 74.78% specificity, and 75.2% accuracy in the second model.

The implementation used in the current study aimed to perform a high-throughput screening of large metagenomic data rather than a limited number of queries. Therefore, defining features for the binary classification of ACP versus non-ACP was selected on the training data (*p* < 0.05). The selected features were then applied to the translated metagenomic data that was generated by 6-frame translations of a sliding window. The sliding window size increased from 5 to 50 amino acids and was derived from the metagenomics reads and assemblies, utilizing standard genetic code. The ACP prediction of the best fitting amino acid and dipeptide composition was further validated using SVM Models [12] against our selection of ACP-specific criteria. It is important to note that the implementation described here did not take into account the amino acids as in the referenced work. However, downstream filtering and annotation scanned for HMM profiles identified in ACPs [22, 23] and, aligned short sequences to the ACPs’ dataset [24], among other criteria described, to select the most likely candidate(s) for experimental validation.

### Metagenomics library screening and candidate peptide selection

We adapted a previously proposed method [12] to search the AUC Metagenomics library for potential anticancer peptide sequences. To compare and select candidate peptides, we examined a dataset of experimentally validated anticancer peptides [19–23, 25] with publicly available dataset of antimicrobial peptides and another of random peptides.

We investigated in a size ranging from 1 to 30 amino acids. Additionally, we also calculated the amino acid and dipeptide (2 amino acids) frequencies from the anticancer and antimicrobial datasets. Using a t-test with Bonferroni multiple testing correction (*p* < 0.05), we compared the frequencies of amino acids and dipeptides in the anticancer and antimicrobial peptide datasets with those in the metagenomic library of peptides. Only peptides that conformed to known amino acid frequencies were selected. A sliding window approach was employed starting from 5 amino acids, to generate peptides from the translated reads of the metagenomics library. The selected peptides, within standard error from the mean, were scored based on their conformity with the mean amino acid and dipeptide frequencies from the experimentally proven anticancer peptide dataset. Furthermore, we analyzed passing peptides for the presence of Hidden Markov Models (HMMs) [24, 26] previously reported on experimentally verified anticancer peptides. Our final predictions were using the online tool developed by Tyagi et al. [12] (http://crdd.osdd.net/raghava/anticp/).

#### Filtering a single candidate for modeling

From the final shortlist of 59 potential anticancer peptides, we selected a single anticancer peptide on criteria such as cationicity, model prediction score, and size.

#### Peptide performance optimization

To enhance the statistical performance of the selected peptide, we compared it against a dataset of experimentally validated and random peptides, and carried out a series of optimization steps. The peptide sequence was submitted as a FASTA file to the AntiCP web server for anticancer peptide prediction. We chose “model 2” analyses, which compared the peptide sequence query to a set of experimentally validated anticancer peptides and random peptides. This model used amino acid frequencies as a predictor for anticancer activity. An unsupervised amino acid reconstruction predictor model was performed on the peptide. Serial modifications were introduced outside the HMM alignment region to maximize the prediction score. Serial modifications were concluded when the prediction model returned a high score to differentiate the query and identified it as an anticancer peptide.

#### Sequence alignments

The two sequence alignments were performed: DELTA-BLASTp (NCBI) and HMMER (EBI). The peptide sequence was queried in FASTA format on the DELTA-BLASTp platform examining the *Homo sapiens* database for all non-redundant proteins with a filter on low complexity regions; while keeping all other parameters set to their default values. We conducted multiple iteration searches until convergence was achieved, where no more new reference sequences aligned with our peptide. Unknown or unnamed sequences were removed in each iteration.

We also used our peptide sequence to conduct an HMMER search against the *Homo sapiens* reference genome, iterating the search until convergence.

#### Predictive modeling

We utilized I-TASSER, a web server for protein structure and function prediction [27]. Output files were extracted for further modeling and analysis. The modeling and visualization software Chimera, developed by UCSF, was used to generate model figures and descriptions of the peptide structure [28]. We also employed the PlifePred web server to predict the estimated blood-borne half-life of our generated peptide sequence [29].

#### Peptide synthesis and preparation

The peptide was synthesized by GL Biochem LTD, Shanghai, China, and obtained as a lyophilized powder with 98% purity at a concentration of 1 mg per vial at -20 °C. For experimental use, a fresh peptide’s stock was prepared by dissolving the 1 mg of lyophilized powder in 1 ml of sterile deionized water, resulting in a concentration of 1 mg/ml.

### Cell culture

The following cell lines were used in this study: SNU449, HepG2, SKOV3, HeLa, and 1BR-hTERT. SNU449 cells were received as a gift from Dr. Mehmet Ozturk at the Department of Molecular Biology and Genetics, Bilkent University, Turkey. HepG2 cells were previously purchased from Vacsera, Egypt, while SKOV3 cells were provided by Dr. Anwar Abdel Nasser, American University in Cairo, Egypt. HeLa cervical adenocarcinoma cells and Immortalized human fibroblast cell line 1BR-hTERT cells [29–31] were provided kindly by Dr. Andreas Kakarougkas, American University in Cairo, Egypt. SNU449 and SKOV3 cells were maintained in complete RPMI-1641 media (Lonza, Verviers, Liege, Belgium), supplemented with 10% heat-inactivated fetal bovine serum (FBS, Gibco, Paisley, Scotland, UK) and 5% Penicillin–Streptomycin (Gibco, Paisley, Scotland, UK). HepG2, HeLa, and 1BR-hTERT cells were kept in DMEM-HG (Lonza, Verviers, Liege, Belgium) completed with 10% heat-inactivated FBS and 5% Penicillin–Streptomycin. Cells were cultured in an incubator at 37 °C with 5% CO_2_.

#### SNU449 doubling time

At three thousand cells/ml, in fresh complete RPMI, the growth rate was calculated at 6 h, 24 h, 48 h, and 72 h. Cells were trypsinized (Trypsin EDTA, 0.25%, phenol red, Lonza, Verviers, Liege, Belgium) for 4—7 min in a humidified incubator set at 37 °C with 5% CO_2_. Trypsin was deactivated with fresh complete RPMI (cRPMI). Cells were collected after addition of cRPMI to deactivate trypsin, via centrifugation at 500 Relative Centrifugal Force (RCF), 4 °C for 5 min. After resuspension in cRPMI, a hemocytometer was used for cell counting using Trypan blue (Lonza, Verviers, Liege, Belgium) staining. 20 μl of cell suspension was added to an equal volume of Trypan blue and loaded into the hemocytometer chambers. The cellular count was performed using the formula:

$$\mathbf T\mathbf h\mathbf e\mathbf o\mathbf r\mathbf e\mathbf m\;1:\frac{cells}{ml}=Cell\;count\times\frac{Dilution\;Factor}{No.\;of\;chambers}\times10,000$$

Cell count was calculated for each time point, and plotted on a growth curve using the following equation (ATCC®):

$$\mathbf{T}\mathbf{h}\mathbf{e}\mathbf{o}\mathbf{r}\mathbf{e}\mathbf{m}\;2:\boldsymbol{ }DT=T\frac{ln2}{ln (\frac{Xe}{Xb})}$$

*DT* is the doubling time.

*T* is the total time during which exponential growth occurs.

*Xb* is the cell number at the beginning of exponential growth.

*Xe* is the cell number at the end of the exponential growth.

#### Cell cytotoxicity assay

SNU449, HepG2, SKOV3, HeLa, and 1Br-hTERT cells were seeded at 5 × 10^3^ cells/well in 96-well plate (Corning, Manassas, VA, USA), in complete fresh media (RPMI/DMEM, 10% FBS and 5% Pen-Strep). All cell lines were treated with the peptide concentration gradient prepared in complete fresh media by serial dilution for an exposure period of 24 h. The peptide concentration gradient for SNU449 cells was constructed for 24 h and 48 h time points and compared to a concentration gradient of cisplatin at 48 h. Treated cells were used to observe morphological changes and then treated with MTT reagent to obtain IC50 calculation.

Peptide-treated media was discarded and replaced with MTT reagent. Stock MTT reagent (5 mg/ml in PBS) (Serva, Heidelberg Baden-Württemberg, Germany) was diluted in complete media to reach a final concentration of 0.5 mg/ml. A diluted reagent was added to all conditions. Cells were incubated in the dark for 2—3 h in a 37 °C, 5% CO_2_ incubator. Media/substrate was discarded and replaced with DMSO (Sigma-Aldrich, St. Louis, MO, USA), added to dissolve formed purple formazan crystals for 15 min in the incubator in complete darkness. Absorbance values were measured at 490 and 570 nm with a BMG Labtech Spectrostar Nano plate reader (Ortenberg, Baden-Württemberg, Germany). Blank corrected absorbance measurements reflected the cell viability percentage, with normalized untreated cell absorbance representing 100% viability.

#### Scratch wound healing assay

A scratch wound healing assay was performed to test the effect of the 37-mer peptide treatment on SNU449 and SKOV3 cell migration ability. SNU449 cells were seeded in duplicate at a density of 2 × 10^5^ cells/well in 12 well plates. SKOV3 cells were plated in 24-well plates (Corning, Manassas, VA, USA), at 5 × 10^4^ cells/well-fed with complete media. Two perpendicular lines were performed using sterile 20 μl pipette tips at 85—90% confluent monolayer cells. Following scratches, cells were gently washed twice with 1X PBS to remove cell debris. Complete RPMI/DMEM media was added to the untreated control, while complete media was supplemented with IC50 of the peptide and added to the treated wells and incubated for 24 h. SNU449 cells were additionally assessed at 48 h treatment with the peptide due to its high calculated doubling time. Images of the scratch were taken at 0 h, 24 h, and 48 h and recorded with an Olympus IX70 inverted microscope. Wound closures were analyzed on ImageJ. The percentage of wound closure was calculated using the following equation:

$$\mathbf{T}\mathbf{h}\mathbf{e}\mathbf{o}\mathbf{r}\mathbf{e}\mathbf{m}\;3:WC\;\%=\frac{wc\;0h-wc\;xh}{wc\;0h }\boldsymbol{ }x 100$$

**WC** % = percentage wound closure (Cellular Migration).

**WC 0 h** = percentage wound closure at zero hours, once the scratch is performed.

**WC xh** = percentage wound closure at a specified time point.

### Reverse Transcription Polymerase Chain Reaction (RT-PCR)

SNU449 and SKOV3 cells treated with the peptide for 24 h were profiled for specific gene expression levels associated with proliferation, epithelial-to-mesenchymal-transition (EMT), apoptosis, autophagy, and survival. Expression levels of *KI67*, *B-catenin*, *Survivin*, *Bax*, *N-cadherin*, *E-cadherin*, *Vimentin* [32], *ATG5*, *ATG6*, and *ATG7* [33] were analyzed using semi-quantitative RT-PCR for the following genes: KI67, B-catenin, Survivin, Bax, N-cadherin, E-cadherin, Vimentin, ATG5, ATG6, and ATG7. Extracted RNA (0.5 ug) was reverse transcribed using RevertAid First Strand cDNA Synthesis Kit (Thermo Fisher Scientific, Vilnius, Vilniaus County, Lithuania), according to the manufacturer’s protocol. The PCR reaction was performed utilizing 1 μL cDNA template in Taq DNA Polymerase (Thermo Fisher Scientific, Vilnius, Vilniaus County, Lithuania) reactions. PCR conditions were standardized for tested genes: 94 °C for 3 min, followed by cycles of 94 °C for 30 s, annealing temperature for 30 s, 72 °C for 45 s, with a final extension carried out at 72 °C for 7 min. Additionally, each gene’s cycle number and annealing temperature were separately adjusted (Table S1) based on optimization conditions. PCR products were run on 2% agarose gel and visualized in a Gel Doc EZ System (Bio-Rad, USA). GAPDH served as an endogenous control.

#### Annexin V apoptosis assay

To further study the peptide cytotoxic effect on the SNU449 cell line, Alexa Fluor 488 annexin V/ Dead Cell kit (Thermo Fisher Scientific, Dartford, England, UK) was used to identify possible cellular death mode. Annexin and PI buffers were prepared according to the manufacturer's instructions. Annexin V, labeled with a fluorophore Alexa Fluor® 488 dye, is a human anticoagulant and has a high binding affinity for phosphatidylserine (PS). PS is exposed on the outer leaflet of the apoptotic cell plasma membrane while presented in the inner membrane of a viable cell. Necrotic cells are dyed with red fluorescence propidium iodide (PI) nucleic acid binding dye. PI can’t bind to apoptotic or viable cells.

SNU449 cells were plated at a density of 3 × 10^5^ per well in 6-well plates. Following 24 h, the media was replaced by the peptide’s IC50 complete media. After incubation, cells were collected via trypsinization. Cells were centrifuged for 5 min at 500 RCF, 4 °C. Cells were resuspended in fresh media. The cellular suspension was mixed with Trypan blue counted via a hemocytometer. The suspension was centrifuged to an equivalent of 0.1 × 10^7^ cells/ml and washed with PBS. Cells were resuspended in 1X annexin binding buffer and incubated for 15 min in complete darkness at room temperature. We visualized the cells on a slide under an Inverted microscope (Olympus IX70, Essex, England, UK), using the appropriate FITC filters.

#### Hemolysis assay

Blood samples were collected from healthy volunteers after acquiring Institutional Review Board (IRB) approval and volunteers signed consent anonymously. Two milliliters of fresh blood was collected for every experiment by a physician in a clinical setting. Human erythrocytes were collected via centrifugation at 1000 × g for 5 min and washed twice with PBS. Anticoagulant EDTA (Ethylenediaminetetraacetic acid) (Sigma-Aldrich, St. Louis, MO, USA) was used to keep erythrocytes dispersed. Following the complete removal of serum, red blood cells were suspended in a 2% PBS solution, where 50 μL of 2% erythrocytes were added to each well of a 96-well plate. Two peptide concentrations were used to test peptide hemolytic activity: IC50 and IC25. 50 μL of each concentration was dissolved in PBS and added to the 2% erythrocytes in the 96-well plate, to reach a final concentration of 1% human erythrocytes in each well. PBS was used as a negative control for hemolysis, while deionized water was used as a positive control. Plates were incubated at 37 °C for 1 h. Following incubation, plates were centrifuged for 10 min at 3000 × g in a plate centrifuge, and the supernatant was transferred to a clean 96-well plate (flat bottom). The release of hemoglobin was detected by measuring the absorbance of the supernatant at 570 nm. Erythrocytes in PBS and deionized water were used as the control group of 0% and 100% hemolysis, respectively. Experiments were performed four times in triplicates.

The equation used to calculate Hemolysis percentage: [34]

$$\mathbf{T}\mathbf{h}\mathbf{e}\mathbf{o}\mathbf{r}\mathbf{e}\mathbf{m}\;4:Hemolysis (\%)=\frac{(Amax-At)}{(Amax-Amin)} \times 100$$

A_max_, A_min_, and A_t_ represented the absorbance values for untreated RBCs, completely hemolised RBCs, and tested RBCs.

### Antimicrobial assay

The antibacterial activity of the peptide was tested on two bacterial strains: *Escherichia coli* DH5-alpha (PTA-4752) (gram-negative strain) and *Staphylococcus aureus* (6538) (gram-positive strain) both ATCC (USA). Overnight culture was prepared in nutrient broth (BD Difco, Berkshire, England, UK) from bacterial glycerol stock and kept in a shaking incubator at 37 °C. 160 μl of the overnight culture was added to 8 ml of fresh media. Optical density (OD) was recorded at different time points, 0, 1, 2, and 3 h, until the reading reached 0.1. At OD = 0.1, the bacteria culture was in the log phase. Serial dilutions were prepared from culture, plated, and incubated overnight. Colonies were counted for 6 dilutions of each bacterial strain for a total of 10^6^ Colony-forming units (CFU). CFU were calculated at OD = 0.1, with no dilutions. Fifty microliters of overnight culture was loaded in 96 well plates, in quadruplets, with 10^6^ CFU in each well. Two peptide concentrations were prepared to be tested: 118.7 μM and 161.6 μM, the median and highest concentrations tested on SNU449. A broad-spectrum antibiotic (Cefoxitin®) was used as a positive control, with a final concentration of 30 mg/ml used in testing. Treated cultures were left overnight in a 37 °C incubator. The effect of the peptide was also assessed as a function of the number of colonies formed in culture (CFU/ml). Following 24-h incubation samples were serially diluted and plated onto agar plates. 20 μl from each condition was used to perform serial dilutions with clean broth and then plated on nutrient agar plates (BD Difco, Berkshire, England, UK) for colony counting. Plates were incubated overnight. Absorbance was read at 600 nm.

Colony-forming unit equation:

$$\mathbf{T}\mathbf{h}\mathbf{e}\mathbf{o}\mathbf{r}\mathbf{e}\mathbf{m}\;5:\frac{CFU}{ml}=\frac{Number\;of\;colonies\times Dilution\;factor}{Culture\;plate\;volume}$$

### Statistical analysis

All data generated was presented as the mean ± standard deviation of three independent experiments, with at least three replicas, and was analyzed using GraphPad Prism 7 software. Multiple comparison analyses were performed using either two-way or one-way ANOVA (analysis of variance) followed by Dunnett's post hoc multiple comparisons test. Pairwise analysis was performed using multiple t-test analysis with multiple comparisons with post hoc correction using the Holm-Sidak method. Dose–response curves and IC50 values were generated in GraphPad Prism 7 using the equation: Log inhibitors vs. Normalized Response-Variable slope *P* values less than 0.05 were considered significant (*P-value < 0.05, **P-value < 0.01, ***P-value < 0.001, ****P-value < 0.0001). R2 values represented the goodness of fit of the non-linear regression model utilized in the cell viability curves. Hill-slope coefficients were also included as an indicator of the cooperativity of ligand-receptor binding, the drug’s affinity to bind to cellular receptors.

## Results

### *In Silico *prediction

#### The most promising peptide candidates contained homeodomains

Fifty-nine candidate anticancer peptides were identified from an SVM search of the AUC metagenomics library. These peptides were classified as either possessing anticancer, antimicrobial, or no activity. Following filtration, all anionic peptides were excluded along with peptides with size larger than 50 amino acids. Two candidate peptides resulted from this filtration process contained HMM alignment profile consistent with homeodomain (PF00046). One Peptide spaned 30 residues while the other spanned 37 residues. The 37-residue peptide was of interest despite the higher cationicity of the shorter peptide, as optimization would alter the homeodomain HMM that spanned the whole length of the 30-residue peptide. The 37-residue peptide was our peptide of interest, with a sequence TKEQKEQIAKATGLTTKQVRNWYVQLNASIKVMLTSI (Table 1 “Original” column) containing a homeodomain HMM profile (PFAM: PF00046.24). This 37-mer peptide was derived from Atlantis II Deep brine pool subsea floor sediment Sect. 6 (available on the NCBI Sequence Read Archive [SRA]). Modification with the p.V32C, p.M33C, and p.S36C amino acid substitutions, which were located outside the homeodomain region, resulted in a final sequence of TKEQKEQIAKATGLTTKQVRNWYVQLNASIKVCMCSC. Peptide sequence modifications increased the peptide's SVM model performance scores and cellular permeability by modifying the ACP/Non-ACP score and the hydrophobicity profile (Table 1 “modified” column).

**Table 1:** Chemical properties of the modified peptides

	**Original**	**Modified**
**Peptide length**	37	37
**HMMid**	Homeobox	Homeobox
**HMM accession**	PF 00046.24	PF 00046.24
**HMM start**	1	1
**HMM end**	31	31
**ACP/NON-ACP score**	-0.98	0.28
**Hydrophobicity**	-0.18	-0.2
**Hydropathicity**	-0.39	-0.34
**Hydrophilicity**	0	-0.01
**Charge**	4	4
**Mol. Weight**	4334.71	4213.53

**Fig. 1:**
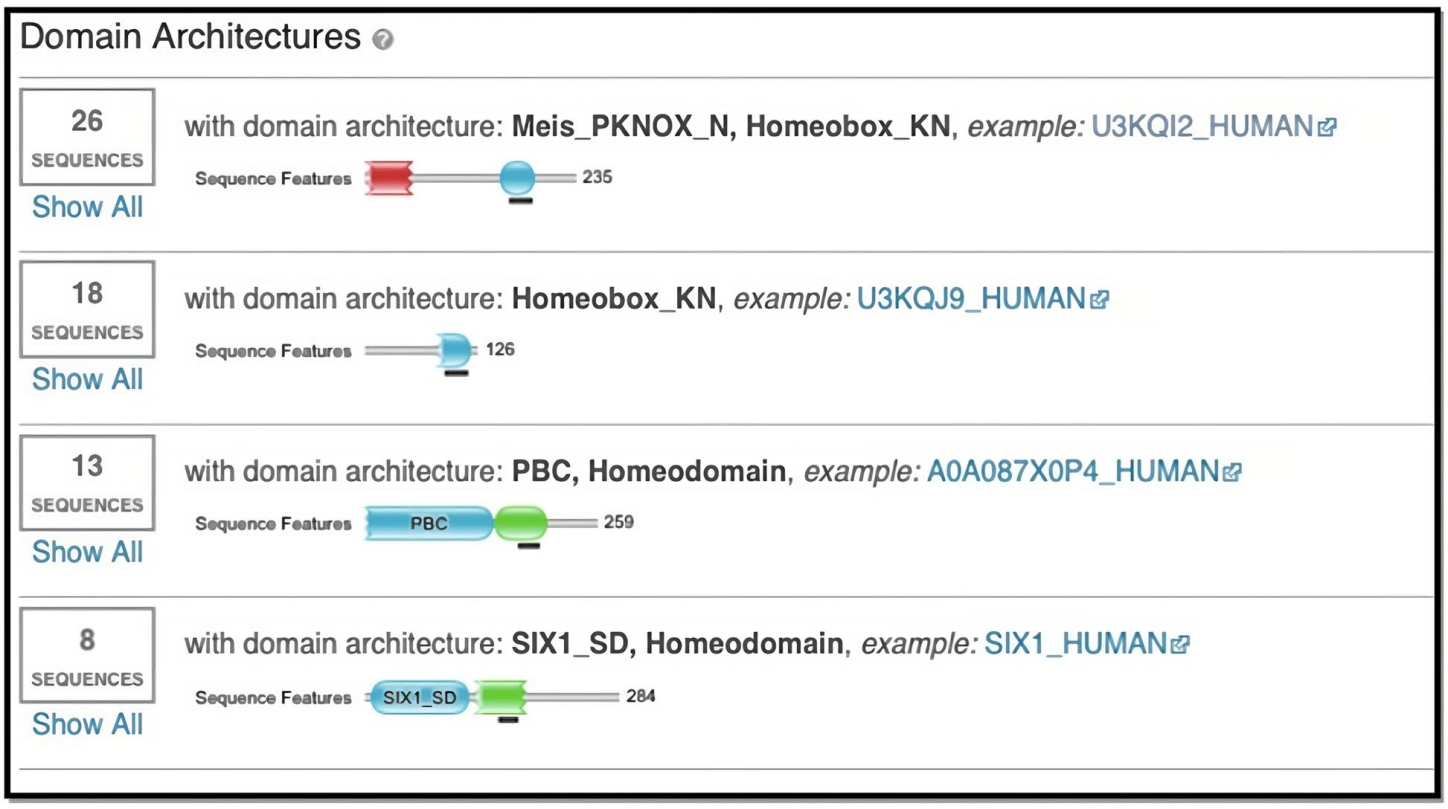
Domain architectures in which the HMMER alignments occurred against the peptide. The alignments occurred within the Meis_PKNOX_N, Hommeobox_KN, PBC, and SIX1_SD domain architectures

An iterative position-specific scoring matrix (PSSM) was used with the NCBI Conserved Domain Database (CDD) as a reference, DELTA-BLASTp alignment revealed that most significant alignments, upon convergence, were with leukemogenic homolog protein (e- value = 2e-7, total score = 45, identity = 45%, cover = 64%) and MEIS2 (e-value = 3e-7, total score = 45, identity = 45%, cover = 64%). Congruent to the BLASTp alignments, HMM-based alignment using EBI’s HMMER identified 65 significant alignments (e-values ranging from 2e-11 to 4.9e-4). Identified alignments were within the homeodomain structure; namely, the homeobox_KN, PBC, and SIX1_SD architectures. The alignment domains indicate similarities with certain protein families such as the MEIS, PBX, and SIX proteins (Fig. 1).

#### The peptide contained helix-coil structure similar to homeoprotein transcription factors

**Fig. 2:**
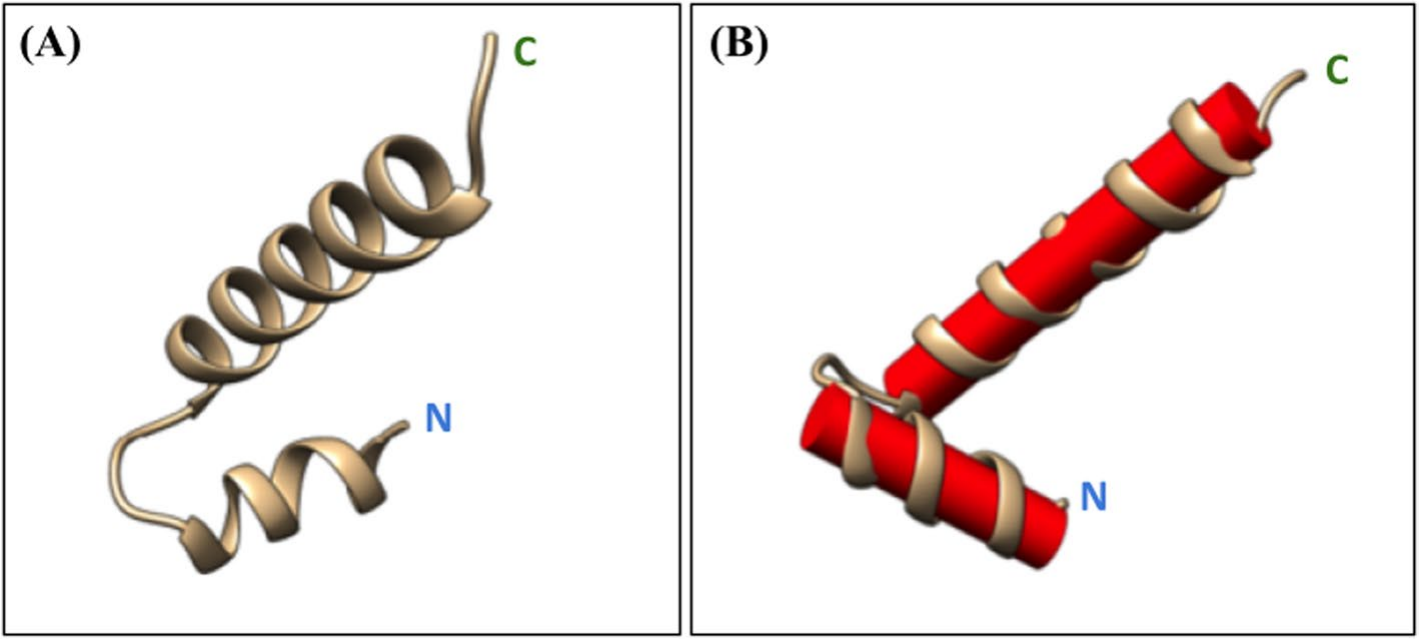
Peptide structure prediction results from I-TASSER. **A** Shows the secondary structure of peptide as predicted by I-TASSER. **B** Shows the angle formed between the axis of the C terminal recognition helix and the N- terminal helix which is about 79 degrees

Inputting the sequence of the 37-mer peptide into I-TASSER structure prediction software revealed a helix-loop-helix structure (Fig. 2A) with a 79° angle tilt between the axis of the C- and N-terminal helices (Fig. 2B). This architecture was consistent with the homeodomain consensus (PFAM).

The DNA-bound structure of the extended PBX homeodomain and MEIS2 were used as templates to predict the structure of the peptide. The homeoprotein transcription factor PAX6 showed the highest structural similarity to our peptide (TM-Score = 0.815, RMSD = 1.39, coverage = 1.000). Gene Ontology (GO) terms were generated for functional prediction by COACH analysis within the I-TASSER suite revealed terms related to sequence-specific DNA binding, noncovalent protein and protein complex binding, regulation of DNA transcription, and nuclear localization (Table 2). Subsequent sequence analysis of the modified peptide predicted a blood borne half-life of 1325.81 s (22.1 min); the original unmodified peptide had a predicted blood half-life of 1567.81 s (26.1 min).

**Table 2:** GO-term annotations and functions predicted by I-TASSER for 37-mer peptide

**GO-term**	**Function**	**Score**
GO:0003700	Sequence-specific DNA-binding transcription factor activity	0.7
GO:0043565	Sequence-specific DNA-binding	0.54
GO:0005515	Protein binding	0.54
GO:0006355	Regulation of transcription	0.7
GO:0005634	Nuclear localization	0.33

### Cell culture analysis

#### MTT assay

**Fig. 3:**
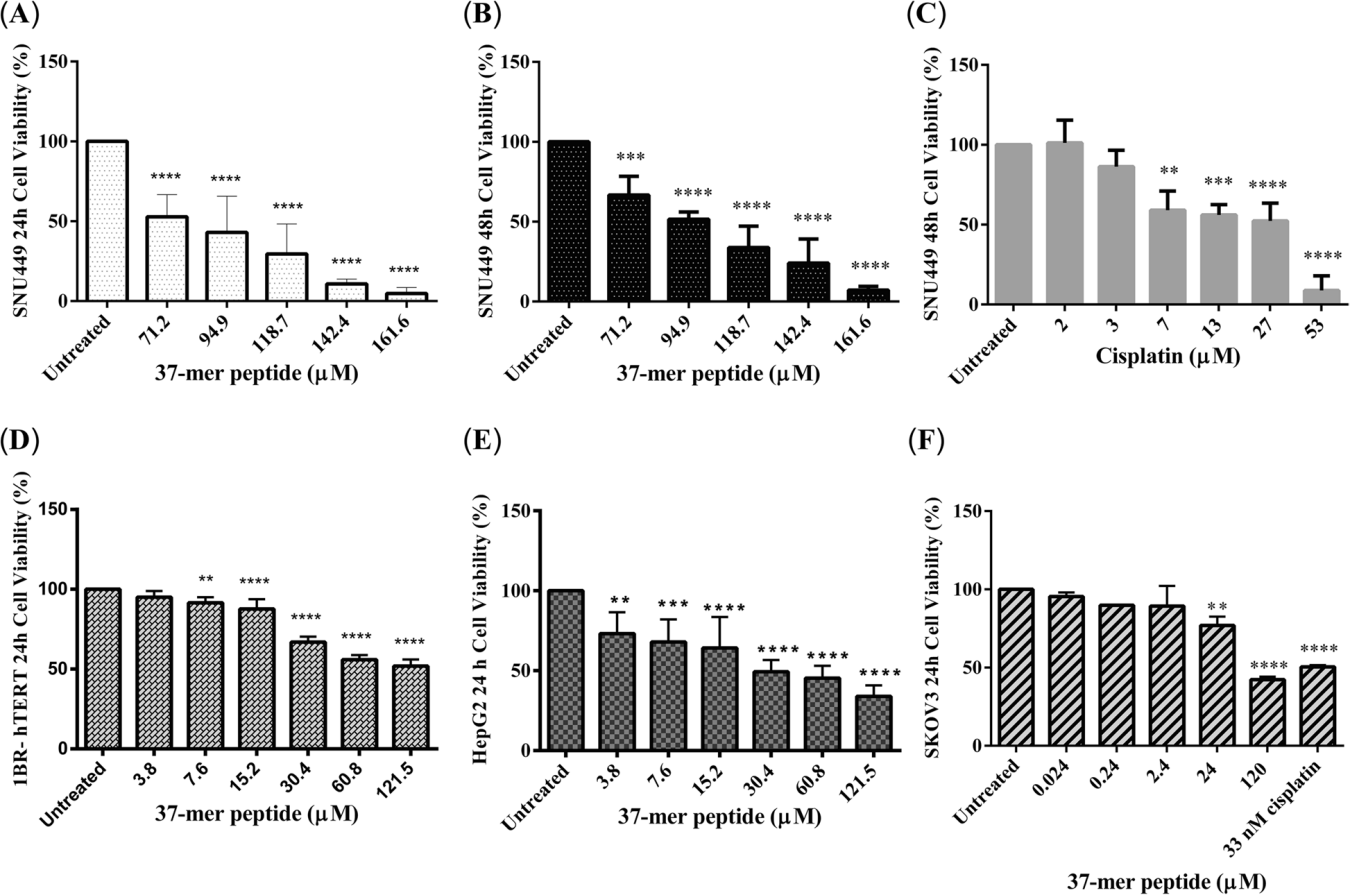
37-mer peptide dose-dependent cytotoxicity. **A** SNU449 displayed a significant decrease in cell viability (approx. 50%) upon treatment with peptide (71.2 μM) at 24 h. **B** Treatment of SNU449 for 48 h with peptide showed a similar trend to 24 h treatment. Cells also experienced significant cell death (approx. 60% viability) at 71.2 μM exposure. **C** SNU449 cells were screened with 48 h treatment of Cisplatin. 7 μM of cisplatin treated SNU449 showed similar cell viability to peptide treatment (71.2 μM). **D** 1BR-hTERT cells were treated with the peptide for 24 h. Cells showed similar viability (approx. 50%) at a lower treatment concentration (60.8 μM) compared to SNU449 cells (71.2 μM). **E** HepG2 peptide treatment at 24 h displayed similar viability (approx. 50%) at a lower concentration (30.4 μM) compared to more advanced hepatocellular carcinoma SNU449 cells (71.2 μM). **F** SKOV3 treated with peptide for 24 h showed a decrease in cell viability (approx. 80%) at 24 μM. Treatment with 33 nM of Cisplatin reduced cell viability by approx. 50%. SKOV3 displayed greater sensitivity to the peptide, as cytotoxicity started to decrease from 0.24 μM of treatment. (** *P* < 0.01, *** *P* < 0.001, **** *P* < 0.0001, *n* = 3)

The MTT assay revealed the 37-mer peptide to exhibit dose-dependent cytotoxicity in SNU449, HepG2, SKOV3, and 1BR-hTERT cells. A gradient of 71 μM to 162 μM was used to assess cytotoxicity of the peptide in SNU449 cells, while a range of 3.8 μM to 121.5 μM was used to examine peptide treatment of HepG2, HeLa, and 1BR-hTERT cells over 24 h. A range of 0.024 μM to 120 μM was used to test SKOV3 cells. For all cell lines, cellular viability decreased significantly with increasing peptide concentration compared with untreated cells (Fig. 3).

The average viability of SNU449 cells treated with 71 μM peptide was 52.8% at 24 h, which dropped to 4.8% after 24 h of treatment with 162 μM peptide. HepG2 cells showed a statistically significant reduction in viability after treatment with the lowest peptide concentration, while SKOV3 displayed greater sensitivity to the peptide, with cytotoxicity observed after treatment with 0.24 μM peptide. Treatment of HeLa cells with 37-mer peptide did not induce a consistent cytotoxic response at any concentration (Figure S1). For 1BR-hTERT cells, a statistically significant drop in cell viability was observed after 24 h incubation with the peptide at concentrations of 15.2 μM and above. Exposure of SNU449 cells to 27 and 53 μM of cisplatin (the positive control) for 48 h caused cell viability to drop significantly to 52.3% and 8.8%, respectively. Treatment with 71 and 162 μM of the peptide resulted in average viability rates of 66.7% and 7.2% respectively.

#### The half-maximal inhibitory concentration

**Fig. 4:**
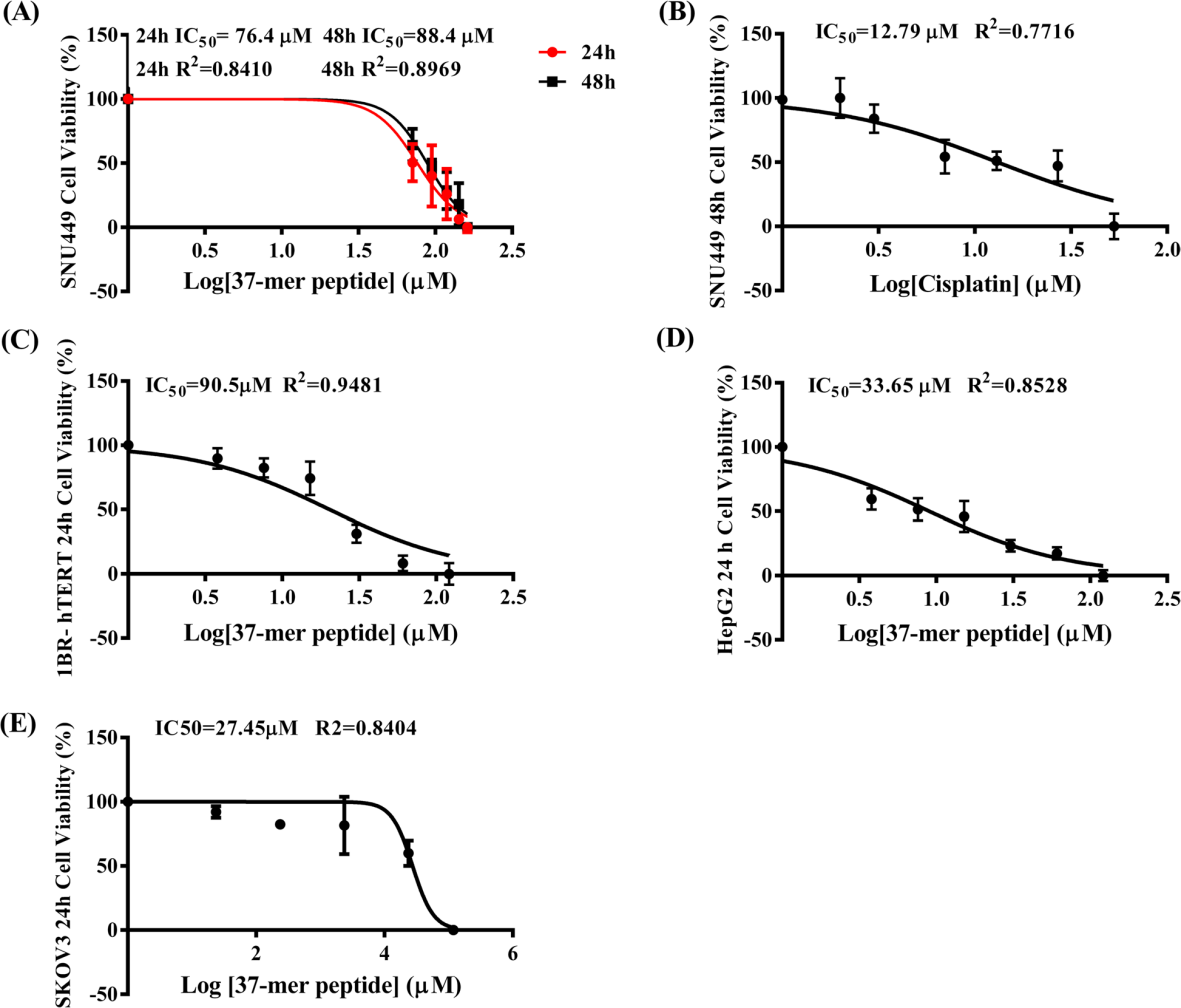
Dose–response of SNU449, 1BR-hTERT, HepG2 and SKOV3, and upon 37-mer peptide treatment. Cell response of each specific cell type was normalized to a scale running from 0 to 100. **A** Peptide treatment response of SNU449. IC50 value was calculated as 76.4 ± 0.6015 μM (R2 = 0.8410) from 24 h treatment, and 88.4 ± 0.9148 μM (R2 = 0.8969), from 48 h exposure, respectively. **B** Cisplatin exposure of SNU449 at 48 h produced a response curve from which the IC50 value was calculated as 12.79 ± 2.055 μM (R2 = 0.7716). **C **1BR-hTERT 24 h dose–response curve generated an IC50 of 90.5 ± 0.521 μM (R2 = 0.9481). **D **HepG2 peptide 24 h treatment response curve produced an IC50 of 33.65 ± 1.09 μM (R2 = 0.8528). **E** SKOV3 response from 24 h peptide treatment produced an IC50 value of 27.45 ± 1.5085 μM (R2 = 0.8404)

The results of nonlinear regression analysis using the MTT data to calculate the relative and absolute IC50, R2, and Hill-slope coefficients of the peptide for SNU499, HepG2, SKOV3, and 1BR-hTERT cells at 24 h time point (Table 3). Dose–response curves for all peptide concentrations were plotted for 24 h and 48 h (SNU449) to calculate IC50 values (Fig. 4). HeLa data are similarly displayed (Table S2).

**Table 3:** Summary of IC50, hill coefficient and R2 values obtained for peptide treatment for all cell lines

**Drug**	**Cell line**	**Time Point (h)**	**Absolute IC50 (μM) ± Std.Error**	**Relative IC50 (μM) ± Std.Error**	**Hill Coefficient ± Std. Error**	**R2**	**Sy.x**
ACP	SNU449	24 h	79.4	76.4 ± 2.6015	3.195 ± 0.6	0.8410	14.59
ACP	SNU449	48 h	93.1	88.4 ± 0.9148	3.56 ± 0.44	0.8969	11.24
Cisplatin	SNU449	48 h	13.7	12.79 ± 2.055	1.183 ± 0.1789	0.7716	16.36
ACP	HepG2	24 h	35.9	33.65 ± 1.09	1.169 ± 0.17	0.8528	18.54
ACP	SKOV3	24 h	28.4	27.45 ± 1.5085	2.67 ± 2.699	0.8404	14.57
ACP	1BR-hTERT	24 h	90.8	90.5 ± 0.521	2.058 ± 0.1958	0.9481	9.03

#### Cell morphology

**Fig. 5:**
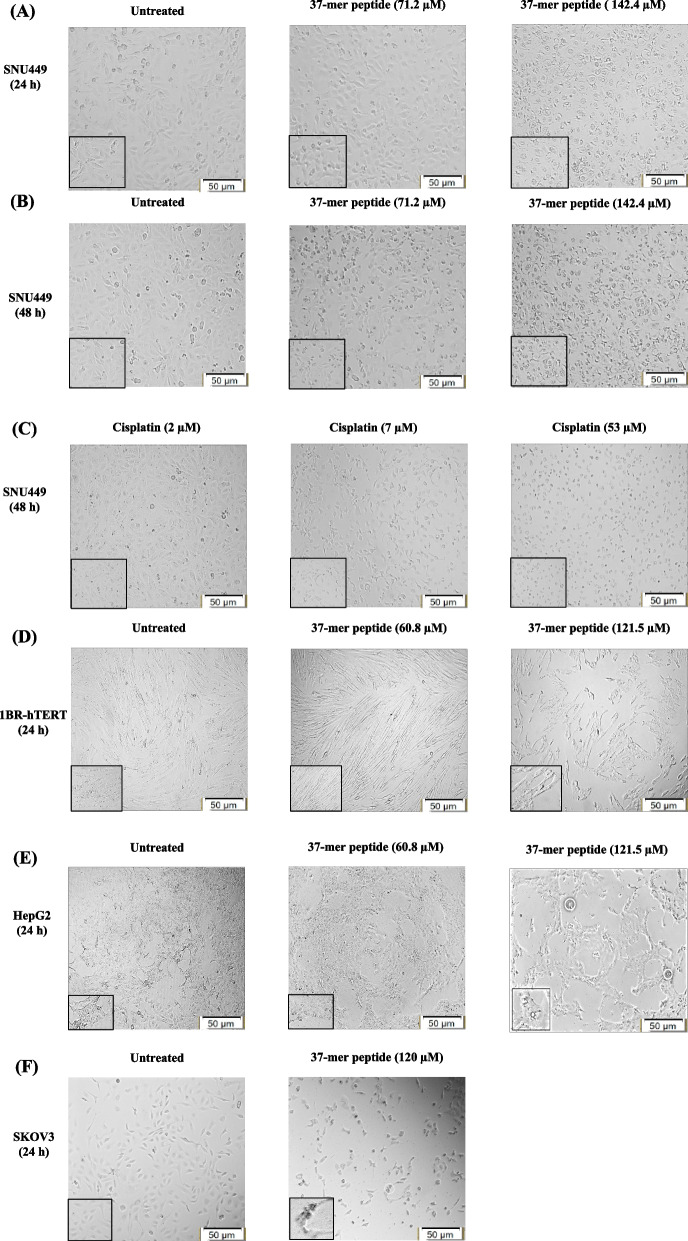
Peptide effect on cellular morphology of SNU449, 1Br-hTERT, HepG2, and SKOV3 cell lines. **A** The lowest (71.2 μM) and one of the highest (142.4 μM) concentrations of peptide treatment on SNU449 cell line at 24 h. Untreated SNU449 cells are compact small spindle-shaped adherent epithelial cells. Treatment with 71.2 μM 37-mer peptide showed cells forming circular shapes and detachment of adherence surface. At higher peptide concentration, cells appeared to be surrounded by debris and form condensed cell detachments. **B **The lowest (71.2 μM) and one of the highest (142.4 μM) concentrations of the peptide on SNU449 cell line at 48 h treatment. Untreated cells in 48 h culture appeared similar to 24 h culture. Treatment with 71.2 μM of the peptide exhibited cells forming rounded sparse distribution. A higher concentration of treatment (142.4 μM) led to the aggregation of ruptured cells forming circular floating detachments. **C** Three concentration points in Cisplatin treatment gradient of SNU449 cells for 48 h: 2 μM, 7 μM, and 53 μM concentration. Induction of 2 μM cisplatin had minimal effect on SNU449 cell morphology compared to untreated cells. 7 μM Cisplatin treatment appeared to halt cell proliferation without changing cellular morphology. High concentration (53 μM) of cisplatin showed excessive cell rounding and reduced cell count. **D** The lowest (60.8 μM) and the highest (121.5 μM) concentrations of peptide treatment on 1BR-hTERT cell line at 24 h. Untreated cells formed elongated, aligned spindle-shaped morphology. The treatment of 1BR-hTERT cells with 60.6 μM appeared to break up cells without causing visible cell death. High concentration (121.5 μM) exposure displayed fragmented cells while losing the spindle elongated morphology, with some cells forming circular shrink appearance. **E** The 2nd highest (60.8 μM) and the highest (121.5 μM) concentrations of 37-mer peptide treatment on HepG2 cell line at 24 h. Untreated HepG2 cells formed a monolayer of an irregular epithelial-like shape. Treatment of HepG2 cells with 60.8 μM caused cells to become sparse, with fragmented growth, while not showing signs of cell rounding. With the highest concentration of treatment, the HepG2 monolayer appeared to be sparse and significantly more fragmented than untreated cells. Additionally, highlighted section included rounded and floating cells, surviving cells do not resemble original cell morphology. **F** SKOV3 cell images of untreated and peptide-treated cells (120 μM). SKOV3 cells formed smaller circular spindle square formations. Administration of 120 μM of peptide caused cells to change morphology and become sparse, circular, and condensed, characteristic of dead cells. The highlighted section shows rounded detached morphed cells with surrounding debris. Cell images are processed at magnification scale of 50 μm

We observed dose-dependent cytotoxicity of SNU449 and HepG2 cells in response to peptide exposure. The epithelial morphology of SNU449 cells transformed into compact round cell structures with vacuoles upon treatment with concentrations of ≥ 142.4 μM resulting in extensive membrane damage, cell rupture, and leakage of cytoplasmic contents (Fig. 5A and B). Cells that remained intact after treatment displayed shrunken cytosolic cell formations (Fig. 5A and B). Most cells appeared viable after treatment with 71 μM peptide, although loss of cellular content was observed. After treatment with cisplatin, SNU449 cells showed circular formations with more noticeable cellular membranes than peptide-treated cells (Fig. 5C). HepG2 cells exhibited fragmentation after treatment with 60.8 μM of the peptide, and treatment with 120 μM resulted in enlarged cells with irregular shapes and rough membranes (Fig. 5D). We also observed the release of multiple large vacuoles from SKOV3, similar to observations with HepG2 cells treated with the highest concentration (Fig. 5E). Treatment of 1BR-hTERT cells resulted in shrunken cells with rough outer membranes compared with untreated cells (Fig. 5F).

#### Scratch-wound healing assay

**Fig. 6:**
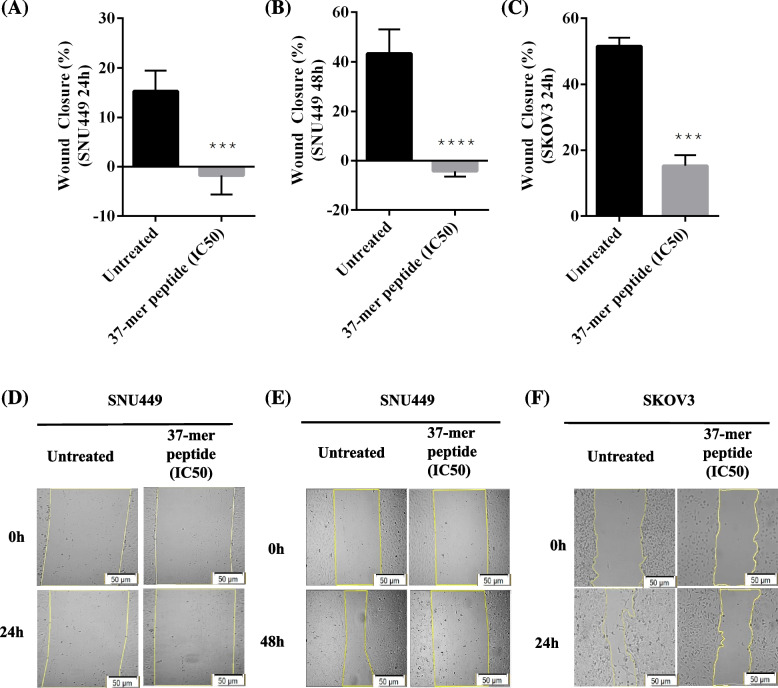
37-mer peptide treatment hindered SNU449 and SKOV3 cell migration. Highly confluent cells were subject to vertical scratches to observe cell migration effect when treated with IC50 of the peptide. **A** and **B** Percentage wound closure of SNU449 cells measured at 24 h and 48 h time points at the IC50 concentration respectively. Wound closure decreased by approximately 20% between untreated and treated (76.4 μM) cells, in addition, the closure also diminished by approximately 50% following 48 h exposure (88.4 μM). **C** Average wound closure of untreated SKOV3s and 37-mer peptide treated (27.5 μM) cells. Treated SKOV3 closure was significantly reduced, by up to 40%, compared to untreated cells (*** *P* < 0.001, **** *P* < 0.0001, *n* = 3). **D** Representative wound closure images of SNU449 cells treated with peptide for 24 h. **E** Similarly, shows wound healing images of SNU449 cells treated for 48 h. **F** Wound closure of SKOV3 cells untreated and treated with peptide. Cell images are processed at magnification scale of 50 μm

The scratch-wound healing assay revealed that the migration of SKOV3 and SNU449 cells was significantly affected by peptide treatment. Exposure of SNU449 cells to peptide treatment for 24 or 48 h resulted in the increase of wound gap by 2% and 4%. In contrast, the wound gap of the untreated cells decreased by 15% and 43% after 24 and 48 h, respectively (Fig. 6). For SKOV3 cells, wound closure of approximately 51.18% was observed for untreated cells but 15.15% for treated cells.

#### Gene expression

Treatment with the peptide for 24 h did not significantly affect gene expression (Figure S2). Peptide treatment caused a significant reduction in *KI67* and *B-catenin* expression with a significant increase in *Vimentin* and *ATG6* expression compared with untreated cells (Figure S3). *ATG7* expression was dampened by peptide treatment of cells. Annexin V apoptosis assay.

**Fig. 7:**
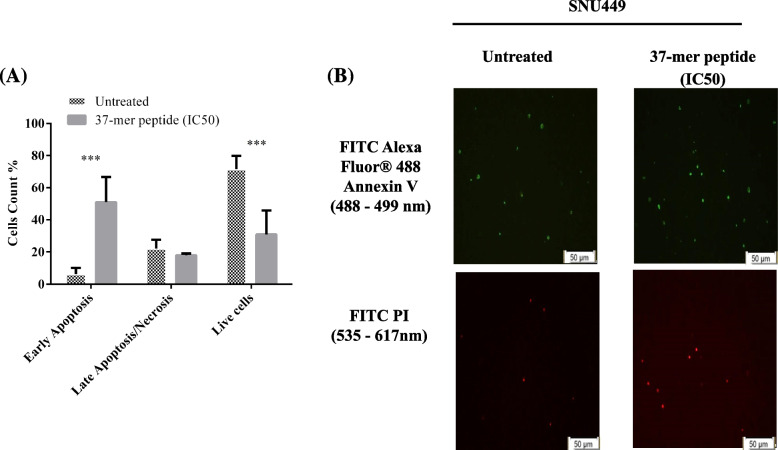
37-mer peptide IC50 treatment apoptotic effect on SNU449 at 24 h. **A** Treated cells exhibited early-stage apoptotic death, no effect on late-stage apoptosis/necrosis, with a significantly lower live cell count (*** *P* < 0.001, *n* = 3). **B** Representative images of annexin V assay microscopy. Merged images showing overlapping green and red signals in untreated control and 37-mer peptide IC50 treated fields. Peptide treatment appeared to increase the rate of early apoptosis in SNU449 cells, compared to untreated cells. Additionally, PI staining was visually similar whether cells were treated or not. Cell images are processed at magnification scale of 50 μm

The annexin V assay revealed that exposure to the peptide caused the number of SNU449 cells early apoptosis to be increased at 24 h compared with untreated control cells (Fig. 7). The proportions of cells that were in early apoptosis, in late apoptosis/necrosis, and live were 51%, 18%, and 31% in the treated cell sample, respectively, compared with 6%, 21%, and 72% in the untreated control.

#### Hemolysis assay

**Fig. 8:**
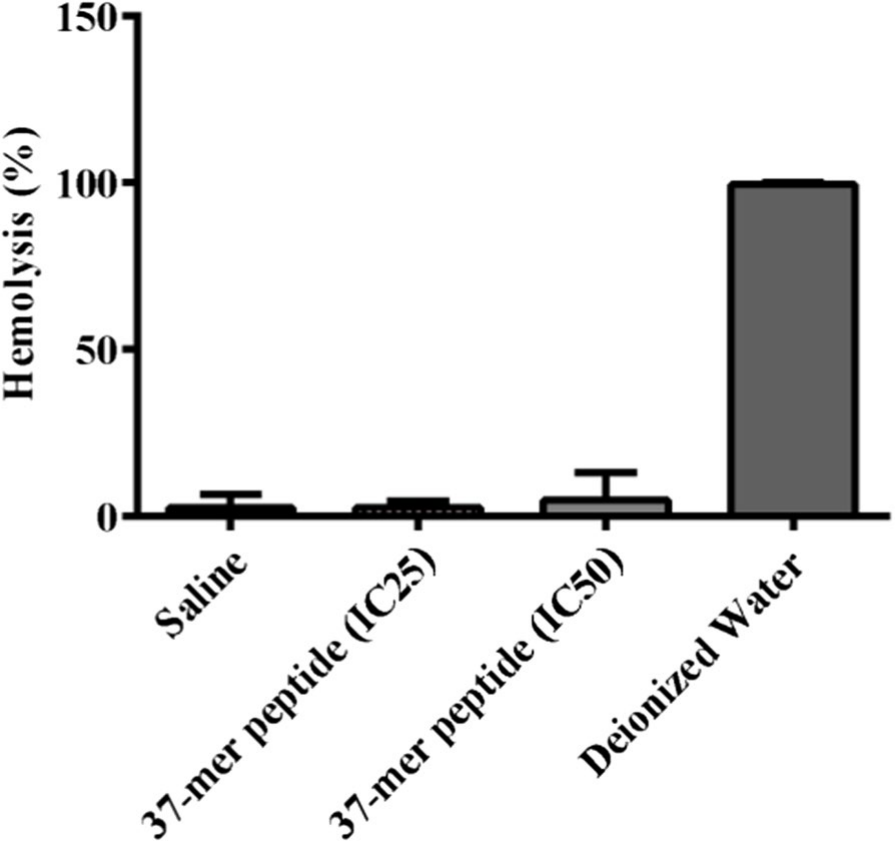
37-mer peptide treatment showed minimal hemolytic activity. The hemolytic activity of SNU449 IC25 (53.8 μM) and IC50 (76.4 μM) concentrations were determined via incubation of erythrocytes for 1 h. Peptide treatment did not show a statistically significant effect on erythrocytes, compared to saline. Deionized water represented standard positive control with 100% hemolysis. Saline represented negative control, *n* = 4

The IC50 and IC25 values calculated from peptide treatment of SNU449 on 2% human erythrocytes (76.4 and 53.8 μM, respectively) were used for the hemolysis assay. Exposure to the peptide at the IC25 and IC50 concentrations resulted in hemolysis of 2.7% and 5% of erythrocytes, respectively, while the saline resulted in hemolysis of 2.7% of erythrocytes (Fig. 8). Normalization of the hemolytic effect of the peptide against the hemolytic effect of saline resulted in the former being deemed negligible at the IC25 and IC50 concentrations.

### Antimicrobial activity assay

**Fig. 9:**
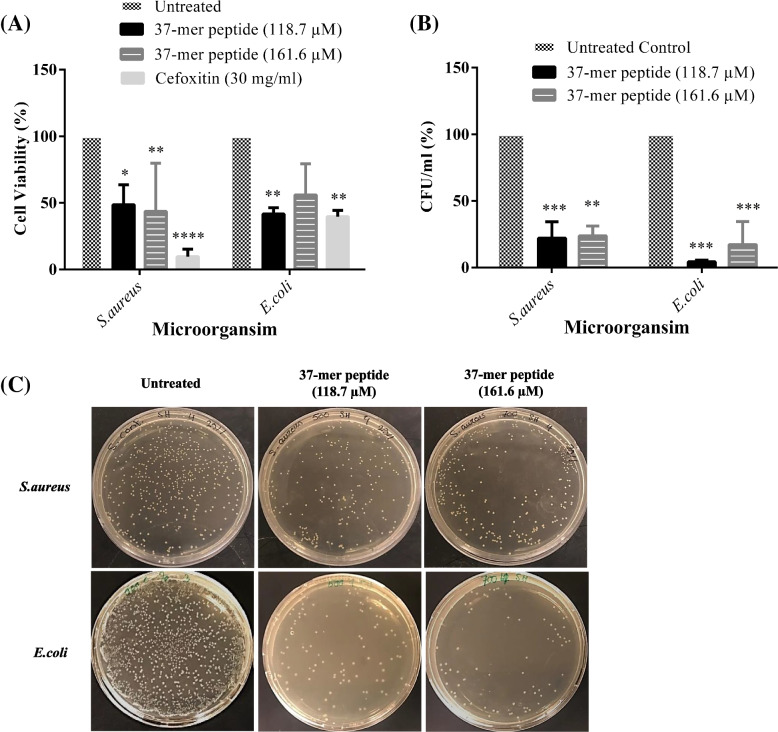
Antimicrobial activity of *S. aureus* and *E. coli* was evident with 37-mer peptide treatment. Statistical representation of peptide antimicrobial activity on S.aureus and E.coli. **A** Effect of 161.6 μM and 118.7 μM of peptide treatment on viable *S. aureus* and *E. coli* bacteria using microdilution assay. Cell viability was determined via absorbance measurements at 600 nm. Cefoxitin (30 mg/ml) was considered a positive control. Peptide treatment resulted in significant loss of viability, approximately 50% or lower, in both gram positive (*S. aureus)* and gram-negative (*E. coli)* bacterial strains. **B** Colony formation unit of *S. aureus* and *E. coli* cells was significantly debilitated (approximately 25%) by both concentrations. *S. aureus* specifically, showed an approximate 20% (118.7 μM) and 25% (161.6 μM) decrease in CFUs overnight exposure, respectively. *E. coli* CFU count displayed an approximate 5% and 15% respective decrease with medium and high treatment, respectively (*** *P* < 0.001, ** *P* < 0.01, *n* = 3). **C** Representative images of agar plates showing viable colonies of *S. aureus* and *E. coli* after overnight culture (10^6^ dilution factor)

The antimicrobial activity of the peptide toward *S. aureus* and *E. coli* at the median and highest concentrations tested (118.7 and 161.6 μM, respectively) were illustrated in (Fig. 9). The turbidity of the samples following treatment indicated that the viability of *S. aureus* dropped significantly to 44% and 49%, respectively, and that of *E. coli* dropped significantly to 56% and 42%, respectively. For comparison, the positive control reduced viability of *S. aureus* and *E. coli* to 10% and 40%, respectively. A broad-spectrum antibiotic, Cefoxitin®, was used as a positive control and applied at 30 mg/ml, caused 10% viability in *S. aureus* and 40% in *E. coli*. Treatment with the peptide caused the number of colonies to decrease significantly for both strains. Following exposure to 118.7 μM and 161.6 μM of peptide, the CFU/ml values for *S. aureus* indicated the level of survival to be 52% and 22%, respectively, of the negative control. For *E. coli*, the level of survival was 20% and 5%, respectively, of the negative control.

## Discussion

We have developed an SVM model to identify anticancer peptides from the AUC Red Sea metagenomics library. We generated a list of 59 potential anticancer peptides, each with a model-performance score, using our SVM. We then selected the best candidate peptide using cationicity and HMM domain as the main criteria and introduced serial modifications outside of the homeodomain region to enhance the model-performance score. The modifications did not significantly decrease the predicted blood-borne half-life.

It is likely that our SVM model did not initially determine any peptides to be potential ACPs because the variations between ACP and non-ACP groups were minimal. Thus, although the model could discern ACPs from AMPs, due to the distinct properties of ACPs, a larger and more diverse dataset was required to produce a clear distinction between these groups and to train the SVM to recognize anticancer peptides from a pool of random peptides.

The DELTA-BLASTp and HMMER alignments showed that our peptide aligned with the homeodomain region of several homeoproteins from the MEIS, PBX, and SIX families, corroborating the previous finding that our peptide contained a homeodomain, suggesting that the peptide would exhibit similar behavior to homeoproteins.

The predicted secondary structure of our peptide revealed two α-helical regions with architecture consistent with the DNA binding region of the homeodomain. Ligand-binding prediction further supported that our peptide would bind to DNA fragments and GO-term annotation implicated the peptide in pathways involving direct sequence-specific binding of DNA and modulation of gene transcription.

The MTT assay revealed our peptide to elicit a cytotoxic response in SNU449, HepG2, and SKOV3 cells. Treatment of HeLa cells caused the formation of numerous large vacuoles, with partial cytotoxicity, while human noncancerous 1BR-hTERT cells exhibited a cytotoxic effect in response to the peptide, although this was attenuated compared with SNU449 or HepG2 cells. The high IC50 value calculated from 1BR-hTERT cell assays demonstrates that our peptide may act selectively toward cancer cells, with a minimal effect on non-cancer cells. The Hill-slope values were all above 1, suggesting that a positive cooperative feedback loop of peptide binding existed.

Cisplatin is an anticancer control drug and was used as a positive control in the present study. The cytotoxic effect of cisplatin toward SNU449 cells was significantly greater than that of our peptide. The IC50 for the SNU449 cells was calculated to be 88.4 μM, compared with 12.8 μM for cisplatin. Considering treatment of SNU449 cells, the higher peptide IC50 at 48 h compared with 24 h suggested that peptide molecules were consumed by cells at 24 h and surviving cells were able to recover and proliferate. Thus, higher concentrations of the peptide would be required to elicit an effective dose–response.

The peptide caused an irreversible morphological change in SNU449, HepG2, SKOV3, and 1BR-hTERT cells after 24 h of exposure. SNU449 cells transitioned from an epithelial-diffuse cell structure to a more compact circular structure, with cells releasing vacuoles resembling autophagosomes and partial rupture and detachment observed. The changes in cellular morphology preceded cell death, which occurred via multiple death pathways. Vacuole formation was also observed in treated HepG2 cells. A major loss in the normal cellular topology was identified in treated cells, most dramatically in SKOV3 cells, which became significantly more fragmented and deformed.

The IC50 values of our peptide were compared to other conventionally used chemotherapeutics. However, chemotherapeutic drugs were extremely cytotoxic to noncancerous drugs; thus, administration at low concentrations was vital to maintain cell survival. Other experimentally validated peptide drugs have shown a wide array of concentrations [35] that also have an antimicrobial slant. Our results have shown that peptide treatment was significantly more effective in stimulating a cytotoxic effect on cancer cells, between 14 – 200% depending on cell type, compared to noncancerous fibroblasts.

A low concentration gradient was tested and optimized on HepG2 cell lines (with IC50 calculated in (Fig. 4). Knowing the invasive properties of SNU449 compared to HepG2 cell lines based on previous studies [36, 37], a higher concentration gradient was developed to characterize the peptide mode of action on SNU449 mesenchymal-like subgroups of hepatocellular carcinoma when compared to HepG2, the less invasive hepatoblast-like subgroup of HCC. Using this higher gradient allowed us to evaluate the peptide cytotoxic effect and potential mode of action. This gradient was also tested on 1Br-hTERT cell lines representative of normal cell lines, showing minimal cytotoxicity.

We determined the primary cell lines of interest based on the peptide dose-dependent response, with the IC50 values of SNU449 and SKOV3 cells being the highest and lowest, respectively, compared with other tested cells. The peptide caused drastic changes in cellular morphology and shrinkage among cells on the sides of the scratches and inhibited SNU449 cell migration, resulting in the enlargement of scratches compared with untreated controls at both 24 h and 48 h of exposure. Additionally, SKOV3 wound closure was severely reduced following peptide treatment. Thus, treatment with the peptide at the IC50 concentration inhibits cellular migration and proliferation of SNU449 and SKOV3 cells with expression of *KI67*, a proliferation marker, decreased in the latter. The unchanged EMT and autophagic genes transcription profiles of SNU449 suggested that changes occurred at the protein level, including epigenetic regulation of these genes [30, 31, 38]. In contrast, expression of *KI67* and *B-catenin* (adhesion marker) was increased in SKOV3 cells following peptide treatment, as was expression of the EMT marker *vimentin* and autophagic markers *ATG5* and *ATG6*. No expression of *ATG7* was detected. These differences may indicate that exposure of SKOV3 cells to the peptide reduced gene expression associated with proliferation, cell adhesion, and autophagy while increasing *vimentin* expression [39]. Further study of the expression of EMT markers and other relevant downstream target proteins is warranted to verify the implications of our findings in relation to the treatment of cancer cells.

Apoptosis is characterized by morphological changes, nucleic acid fragmentation, and loss of membrane asymmetry [40]. The annexin V assay revealed a clear bias toward early apoptosis for peptide treated SNU449 cells, and found to be highly presented mode of cell death upon peptide treatment. In treated cells, 51% of cells were in early apoptosis in comparison to the untreated control displaying only 6% of the cells in early apoptosis, while 18% of the cells were in late apoptotic/necrotic. This indicated that peptide treatment had high percentage of different stages of apoptosis, suggesting apoptosis as an important cellular death pathway to further explore. The differences between the fields of untreated control and treated cells show that peptide treatment drives cells into apoptotic programmed cell death after 24 h exposure, due to cellular presentation of phosphatidylserine (PS) on the outer plasma membrane. Impact of peptide treatment on cellular viability, morphology and migration; demonstrated anticancer properties on SNU449 cells. The proportion of cells in each cellular death phase, early and late apoptosis, confirmed that apoptosis was the most prevalent mode of cellular death following treatment [41].

Hemolytic analysis indicated that our novel peptide did not cause normal erythrocytes to rupture during the incubation period, in agreement with other reports of similar peptides [42], suggesting that the peptide’s cytotoxicity was higher in relatively anionic cancer cells than normal neutral cells. Therefore, we propose intravenous administration would be appropriate for in vivo investigations. The blood-borne predicted half-life of the peptide is similar to the 20—30 min half-life of the platinum-based drug, cisplatin [43], indicating that it would persist within the bloodstream. The Hemolysis Assay was used to validate the potential application of the peptide in vivo through intravenous application and subsequently identify itseffect on RBCs as representatives for normal primary cells. Thr IC50 and the IC25 showed negligible cytotoxic effects of the peptide (Fig. 8). The promising results from cell viability, proliferation, morphology, and migration analyses, and the minimal hemolytic activity of our peptide support its application as a novel anticancer therapeutic [44].

Antimicrobial peptides (specifically, host defense peptides) are part of natural host defense mechanisms against pathogens in the innate immune system of all species [39, 40, 45]. Most previously studied AMPs elicit broad-spectrum effects on all microbes including bacteria, viruses, and fungi [10]. The antibacterial effects of our novel 37-mer peptide toward *S. aureus* [46] and *E. coli* [47] which are part of the normal human flora, colonizing the skin and most of the mucosal membrane microbiota and the gastrointestinal tract, respectively [41, 42] highlight a potential consequence of the peptide upon administration to humans. However, the IC50 of our peptide was much lower in all tested cell lines than the concentrations we tested on both bacterial strains; thus use of the peptide as a therapeutic agent may have a marginal effect on the normal flora. The negligible hemolytic activity of the peptide toward normal erythrocytes suggests that intravenous administration would be the preferable mode of administration.

## Conclusion

We present the identification and characterization of a novel peptide with dual antimicrobial and anticancer properties. The peptide has potential to be a promising anticancer therapeutic agent with rapid and targeted action, with potential to prevent cancerous cells from acquiring metastatic properties [10]. Peptide drugs represent a novel exploratory therapeutic for cancer research, with the potential to overcome the limitations of existing therapies. Our novel antimicrobial peptide exhibits a preliminary anticancer effect on hepatocellular carcinoma cells and ovarian cancer cells, with moderate activity toward immortalized mammalian fibroblasts. Cells treated with the peptide showed morphological degeneration, inhibited migration, early apoptotic cell death, and vacuole formation. In contrast, limited cytotoxicity was observed in erythrocytes. Optimization of the electrostatic and structural properties of our peptide, through sequence modifications, significantly increased its anticancer profile. The dual properties of the peptide against cancer cells and bacterial strains support the peptide’s quick mode of action, as a promising anticancer therapeutic agent, hindering cancerous cells from acquiring resistance. Future studies of our anticancer peptide using an animal cancer model or more physiologically relevant 3D tumor models are warranted.

## Supplementary Information

**Additional file 1: Figure S1. **Effect of 37-mer peptide treatment on HeLa cells over 24 h. **A** Data showed negligible effect on cell viability with an increase in peptide concentration (** *P* < 0.01, *** *P* < 0.001, *n*=3). **B** Treatment of HeLa cells with 121.5 µM. Untreated cells formed compact circular condensed attached cells. Peptide treatment caused cell morphology to become more rounded, sparse, and detach from the plate. Highlighted circle regions showed rounded dead cells releasing cellular vacuoles. Cell images are processed at magnification scale of 50 µm.

**Additional file 2:** **Figure S2. **Gene expression of SNU449 cells of certain Epithelial to Mesenchymal markers and Autophagy genes was not affected by peptide treatment. (A) Gene expression level of *KI67, B-Catenin, Survivin, Bax, N-cadherin, E-cadherin, and Vimentin *from SNU449 24 h treated (IC50) and untreated cells (*n*=3). Gene expression data were generated and normalized against *GAPDH* as a control. Expression data did not statistically change between untreated and treated cells. (B) Autophagy gene expression of SNU449 cells from *ATG5, ATG6, and ATG7 *was determined. Treated cells did not display a statically significant variation in expression in epithelial to mesenchymal transition genes and autophagy genes.

**Additional file 3:** **Figure S3. **37-mer peptide treatment affected gene expression levels of certain EMT and Autophagy markers of SKOV3 cells. (A) SKOV3s were treated with peptide IC50 for 24 h and profiled for EMT and Autophagy markers. Gene expression data were generated and normalized against *GAPDH* as a control. *KI67, B-Catenin*, and *Vimentin *differential expression were significant in treated cells (*KI67 *and* B-Catenin*decreased, while *Vimentin* increased) compared to control. (B) Expression of Autophagy genes showed a significant increase of *ATG5 *and *ATG6* treated cells, compared to untreated SKOV3s, while exposure to the peptide inhibited expression of *ATG7* compared to untreated cells (“**” denotes *P*<0.001, “****” denotes *P*<0.00001, *n*=3).

**Additional file 4:** **Table S1. **RT-PCR primer parameters: sequences, annealing temperature, cycle number, and amplicon size.

**Additional file 5:** **Table S2. **Summary of IC50, hill coefficient, and R2 values obtained for peptide treatment of HeLa and MCF7 cell lines.

**Additional file 6:** **Table S3. **List of sediments used for Biosamples collection with QC values calculated post quality control for raw sequenced reads. Data available in NCBI SRA under PRJNA299097 and PRJNA193416.

## Data Availability

The methods utilized for scanning peptides with potential anticancer activity on a large scale dataset from AUC/KAUST Red Sea Microbiome Project was adapted from the work described by Tyagi, Atul, et al. 2013 [12], where a set of experimentally proven anticancer peptides (ACPs) from antimicrobial databases was compared to another set of antimicrobial peptides (AMPs) with no anticancer activity. Databases used during in silico prediction. 1. Anticancer peptides (ACP) dataset of 225 experimentally validated anticancer peptides from antimicrobial database (APD3, https://aps.unmc.edu/database/anti), collection of antimicrobial peptides (CAMP, http://www.bicnirrh.res.in/antimicrobial), and database of anuran defense peptides (DADP, http://split4.pmfst.hr/dadp/). 2. Antimicrobial peptides with no anticancer activity (AMP_neg) dataset of 225 randomly selected peptides from APD, CAMP and DADP databases. Biosamples adopted from the AUC/KAUST Red Sea Microbiome Project, that are used in the prediction model are uploaded on NCBI with accession numbers as follows: 1. AUC-KAUST Red Sea Sediment Metagenome Bioproject (PRJNA299097). SAMN04193509, SAMN04193508, SAMN04193507, SAMN04193506, SAMN04193505, SAMN04193504, SAMN04193503, SAMN04193502, SAMN04193501, SAMN04193500, SAMN04193499, SAMN04193498, SAMN04193497, SAMN04193496, SAMN04193495, SAMN04193494 2. AUC-KAUST Red Sea Water column and Brine Metagenome Bioproject (PRJNA193416). SAMN03983350, SAMN03983347, SAMN02401045, SAMN02401044, SAMN02401043, SAMN02401042, SAMN02401041, SAMN02401040, SAMN02401039, SAMN02401038, SAMN02401037, SAMN01984728 The biological activity data used to support the findings of this study are included both within the article and in the supplementary material. Inquiries regarding raw data used in this study can be made through the corresponding author, Asma Amleh; aamleh@aucegypt.edu.

## Abbreviations

kDa: Kilodalton
SVM: Support vector machine
AUC: American University in Cairo
SNU449: Hepatocellular carcinoma, grade II-III/IV cell line
HepG2: Hepatocellular carcinoma cell line
SKOV3: Ovarian adenocarcinoma cell line
HeLa: Cervical carcinoma cell line
MTT-assay: 3-[4,5-Dimethylthiazol-2-yl]-2,5 diphenyl tetrazolium bromide
1Br-hTERT: Human fibroblast cell line
ACP: Anticancer peptides
AMP: Antimicrobial peptides
S. aureus: Staphylococcus aureus
E.coli: Escherichia coli
HMM: Hidden markov model
RCF: Relative centrifugal force
RT-PCR: Reverse transcription polymerase chain reaction
PS: Phosphatidylserine
PI: Propidium iodide
IC50: Half maximal inhibitory concentration
IRB: Institutional Review Board
RBCs: Red blood cells
OD: Optical density
CFU: Colony forming unit
CDD: Conserved domain database
GO-term: Gene ontology term
KAUST: King Abdullah University of Science and Technology
