# Supplementary material for: Characterization of a novel peptide mined from the Red Sea brine pools and modified to enhance its anticancer activity
Source: BMC Cancer. 2023 Jul 26;23:699. doi: 10.1186/s12885-023-11045-4 (PMC10369728; doi:10.1186/s12885-023-11045-4)
Supplement: Supplementary file 1 — Additional file 1: Figure S1. Effect of 37-mer peptide treatment on HeLa cells over 24 h. A Data showed negligible effect on cell viability with an increase in peptide concentration (** P < 0.01, *** P < 0.001, n=3). B Treatment of HeLa cells with 121.5 µM. Untreated cells formed compact circular condensed attached cells. Peptide treatment caused cell morphology to become more rounded, sparse, and detach from the plate. Highlighted circle regions showed rounded dead cells releasing cellular vacuoles. Cell images are processed at magnification scale of 50 µm. [file 12885_2023_11045_MOESM1_ESM.pptx]

## Slide 1
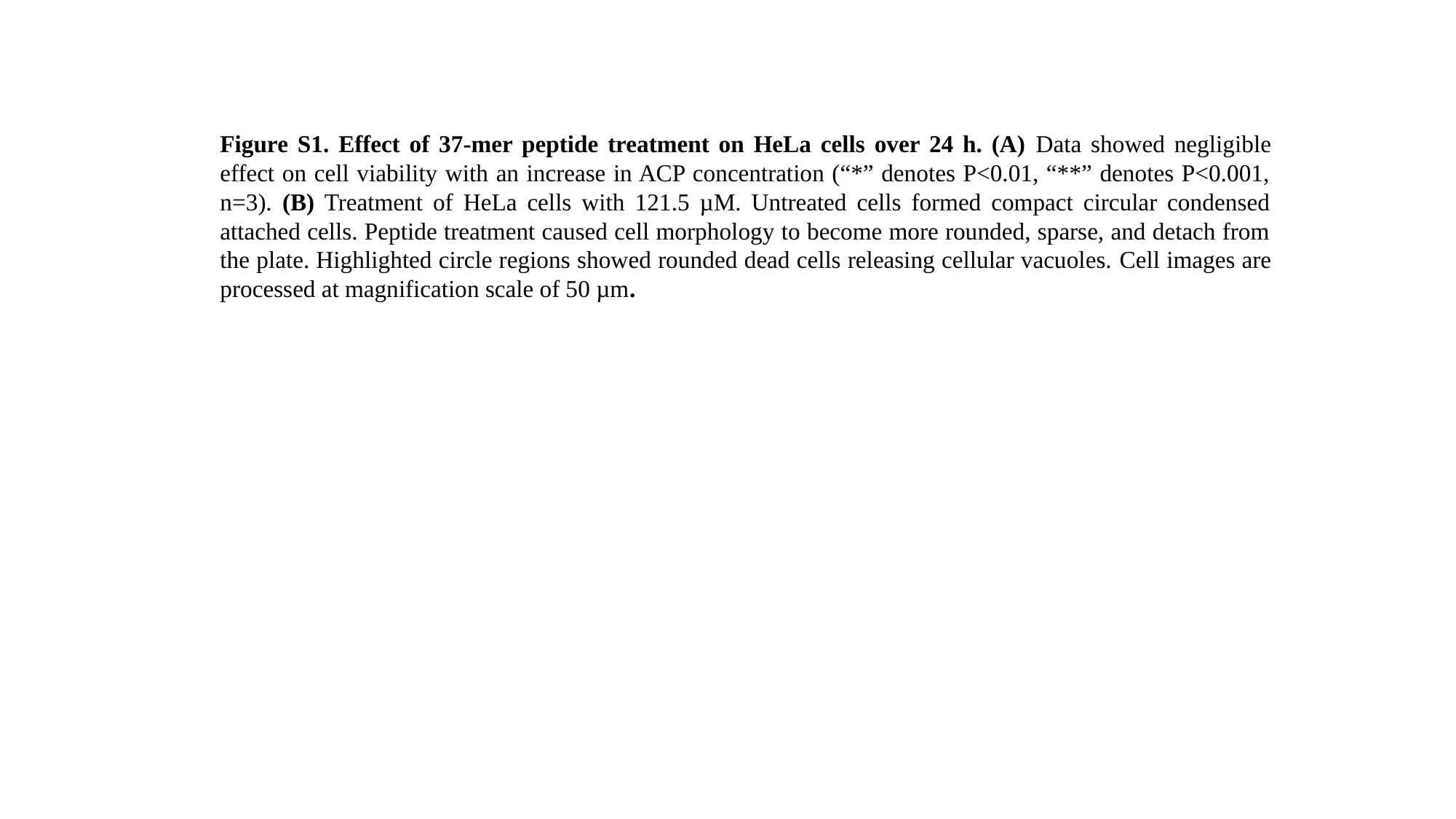

Figure S1. Effect of 37-mer peptide treatment on HeLa cells over 24 h. (A) Data showed negligible effect on cell viability with an increase in ACP concentration (“*” denotes P<0.01, “**” denotes P<0.001, n=3). (B) Treatment of HeLa cells with 121.5 µM. Untreated cells formed compact circular condensed attached cells. Peptide treatment caused cell morphology to become more rounded, sparse, and detach from the plate. Highlighted circle regions showed rounded dead cells releasing cellular vacuoles. Cell images are processed at magnification scale of 50 µm.

## Slide 2
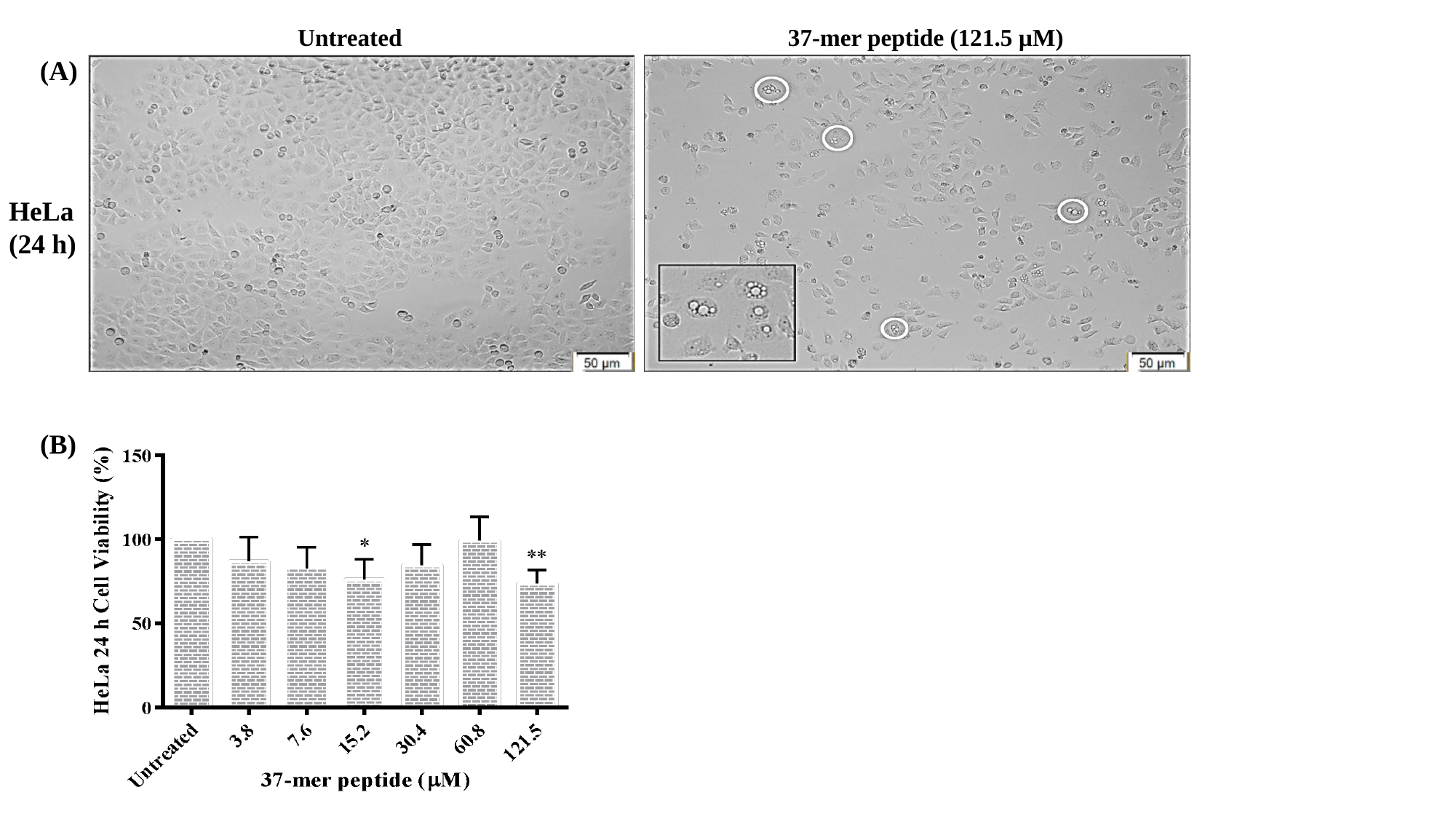

Untreated
37-mer peptide (121.5 µM)
(A)
HeLa
(24 h)
(B)
