# Supplementary material for: Characterization of a novel peptide mined from the Red Sea brine pools and modified to enhance its anticancer activity
Source: BMC Cancer. 2023 Jul 26;23:699. doi: 10.1186/s12885-023-11045-4 (PMC10369728; doi:10.1186/s12885-023-11045-4)
Supplement: Supplementary file 2 — Additional file 2: Figure S2. Gene expression of SNU449 cells of certain Epithelial to Mesenchymal markers and Autophagy genes was not affected by peptide treatment. (A) Gene expression level of KI67, B-Catenin, Survivin, Bax, N-cadherin, E-cadherin, and Vimentin from SNU449 24 h treated (IC50) and untreated cells (n=3). Gene expression data were generated and normalized against GAPDH as a control. Expression data did not statistically change between untreated and treated cells. (B) Autophagy gene expression of SNU449 cells from ATG5, ATG6, and ATG7 was determined. Treated cells did not display a statically significant variation in expression in epithelial to mesenchymal transition genes and autophagy genes. [file 12885_2023_11045_MOESM2_ESM.pptx]

## Slide 1
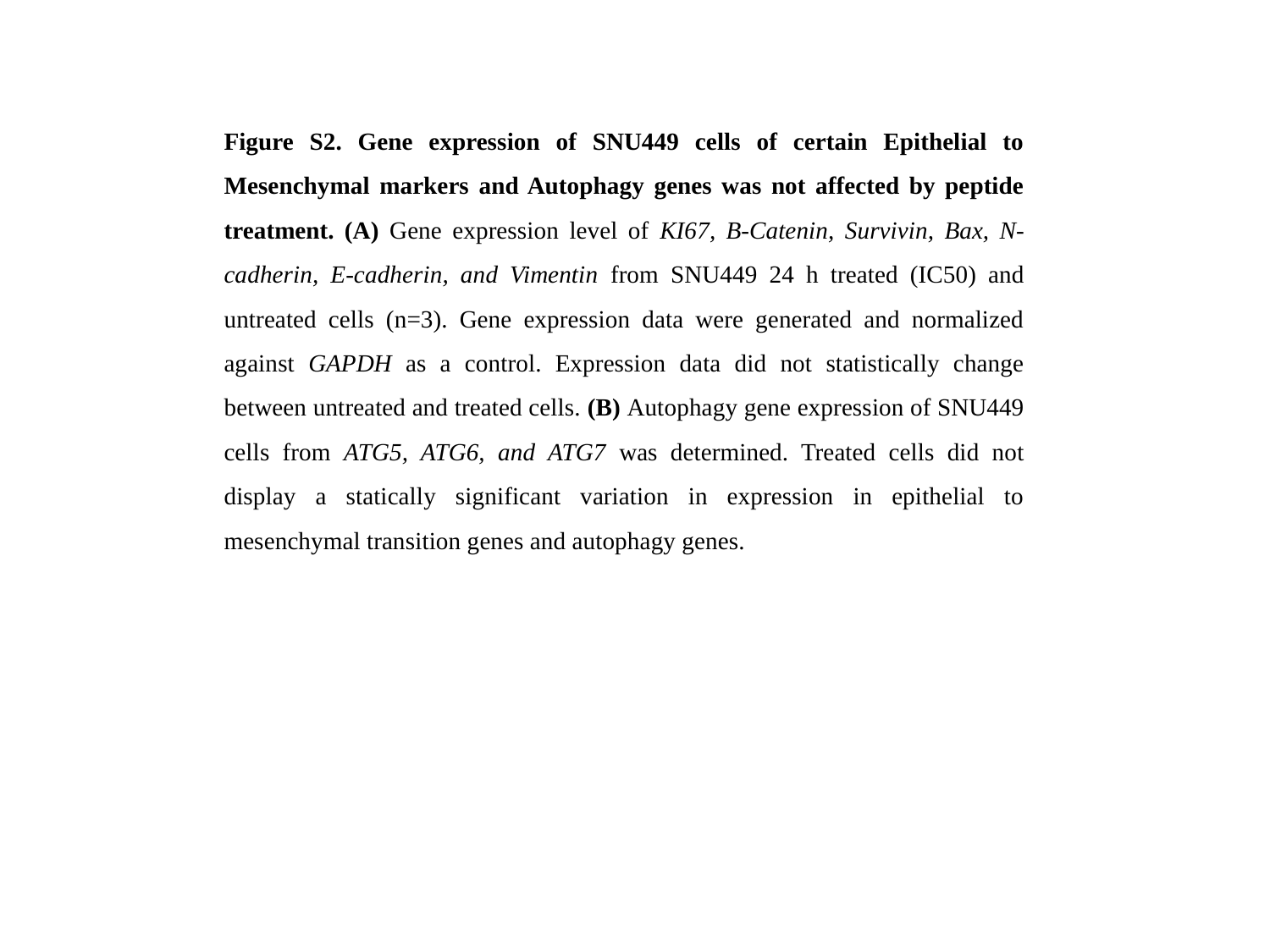

Figure S2. Gene expression of SNU449 cells of certain Epithelial to Mesenchymal markers and Autophagy genes was not affected by peptide treatment. (A) Gene expression level of KI67, B-Catenin, Survivin, Bax, N-cadherin, E-cadherin, and Vimentin from SNU449 24 h treated (IC50) and untreated cells (n=3). Gene expression data were generated and normalized against GAPDH as a control. Expression data did not statistically change between untreated and treated cells. (B) Autophagy gene expression of SNU449 cells from ATG5, ATG6, and ATG7 was determined. Treated cells did not display a statically significant variation in expression in epithelial to mesenchymal transition genes and autophagy genes.

## Slide 2
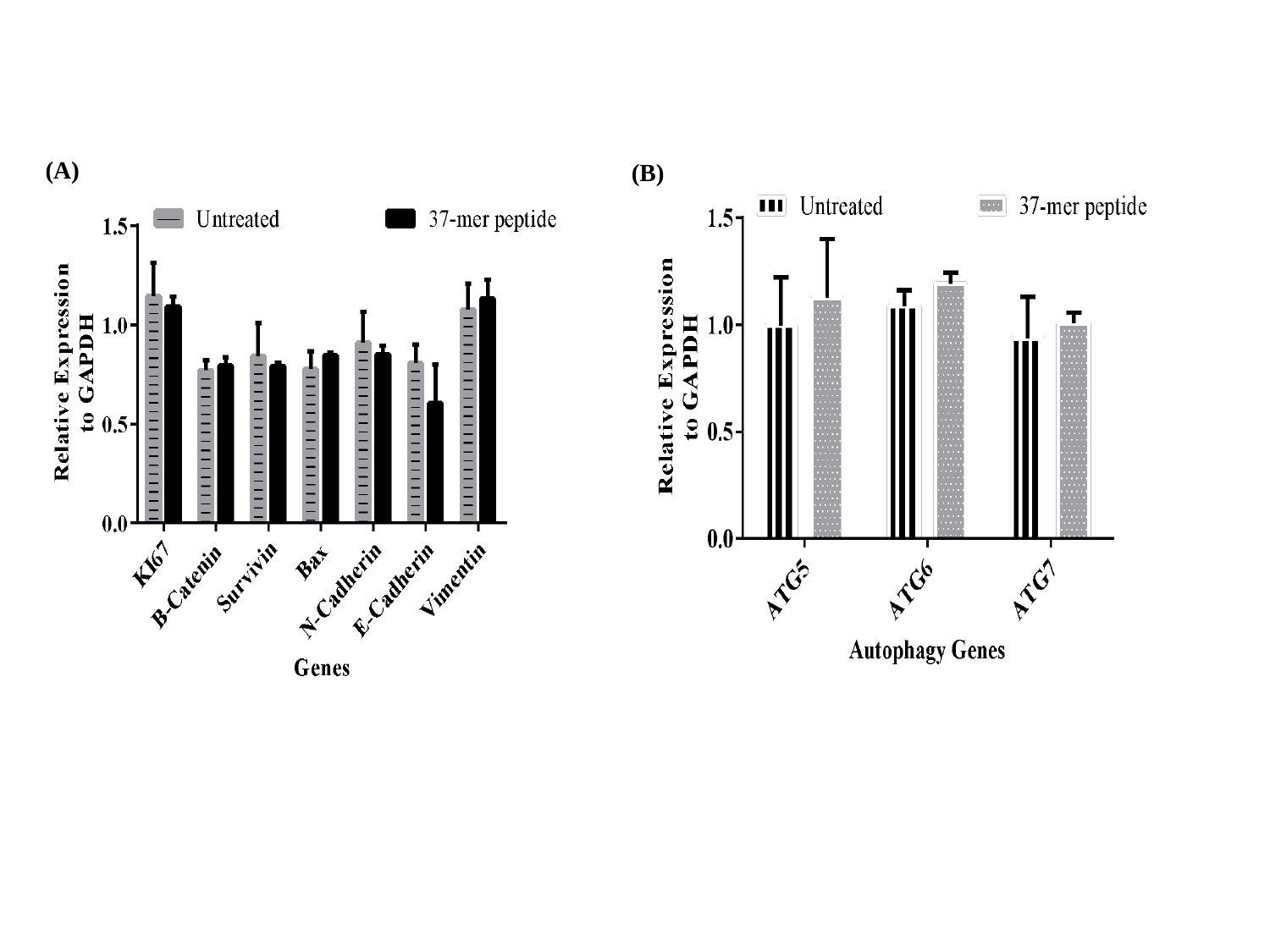

(A)
(B)
