# Supplementary material for: Characterization of a novel peptide mined from the Red Sea brine pools and modified to enhance its anticancer activity
Source: BMC Cancer. 2023 Jul 26;23:699. doi: 10.1186/s12885-023-11045-4 (PMC10369728; doi:10.1186/s12885-023-11045-4)
Supplement: Supplementary file 3 — Additional file 3: Figure S3. 37-mer peptide treatment affected gene expression levels of certain EMT and Autophagy markers of SKOV3 cells. (A) SKOV3s were treated with peptide IC50 for 24 h and profiled for EMT and Autophagy markers. Gene expression data were generated and normalized against GAPDH as a control. KI67, B-Catenin, and Vimentin differential expression were significant in treated cells (KI67 and B-Catenindecreased, while Vimentin increased) compared to control. (B) Expression of Autophagy genes showed a significant increase of ATG5 and ATG6 treated cells, compared to untreated SKOV3s, while exposure to the peptide inhibited expression of ATG7 compared to untreated cells (“**” denotes P<0.001, “****” denotes P<0.00001, n=3). [file 12885_2023_11045_MOESM3_ESM.pptx]

## Slide 1
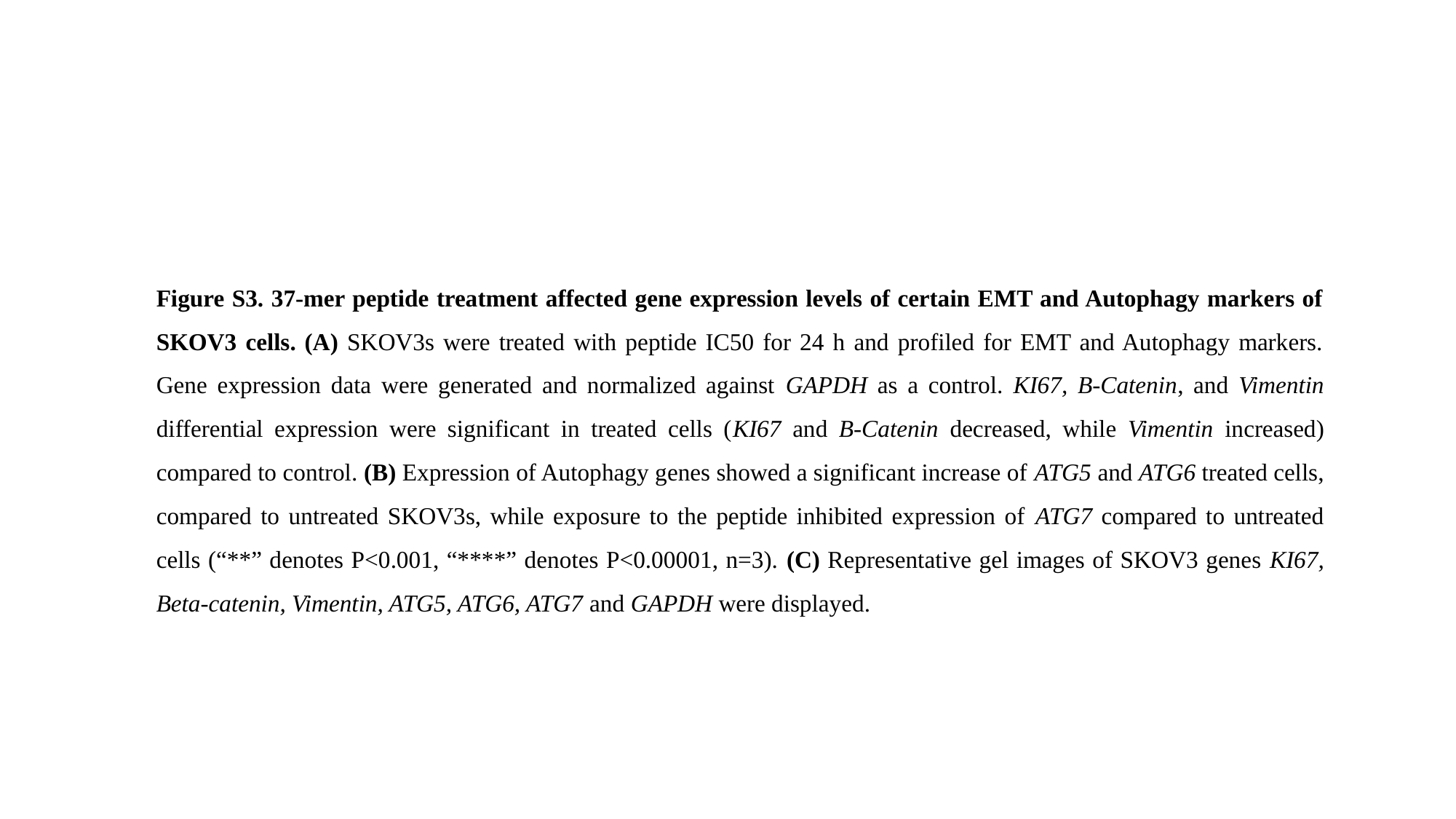

Figure S3. 37-mer peptide treatment affected gene expression levels of certain EMT and Autophagy markers of SKOV3 cells. (A) SKOV3s were treated with peptide IC50 for 24 h and profiled for EMT and Autophagy markers. Gene expression data were generated and normalized against GAPDH as a control. KI67, B-Catenin, and Vimentin differential expression were significant in treated cells (KI67 and B-Catenin decreased, while Vimentin increased) compared to control. (B) Expression of Autophagy genes showed a significant increase of ATG5 and ATG6 treated cells, compared to untreated SKOV3s, while exposure to the peptide inhibited expression of ATG7 compared to untreated cells (“**” denotes P<0.001, “****” denotes P<0.00001, n=3). (C) Representative gel images of SKOV3 genes KI67, Beta-catenin, Vimentin, ATG5, ATG6, ATG7 and GAPDH were displayed.

## Slide 2
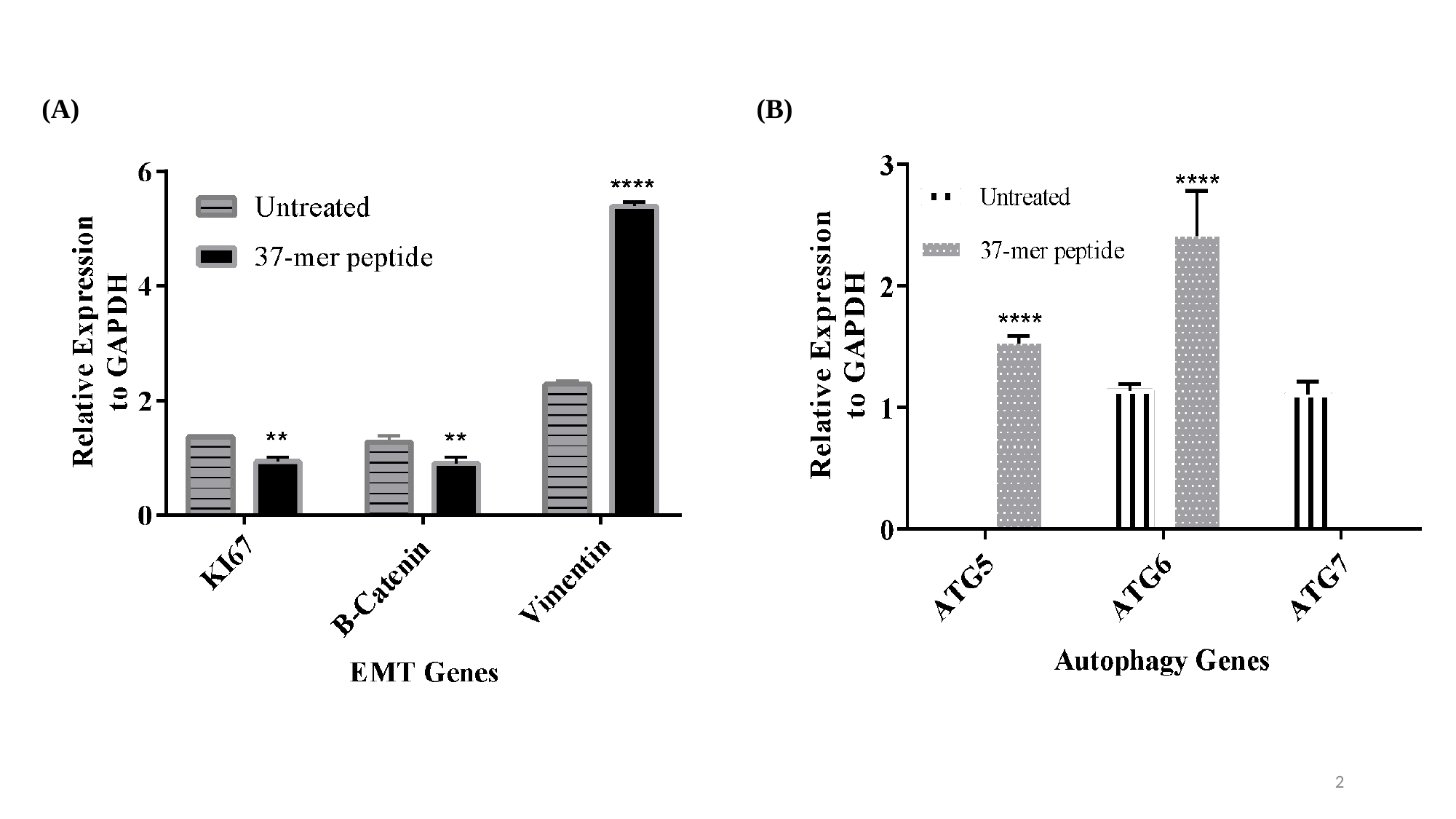

(A)
(B)
2

## Slide 3
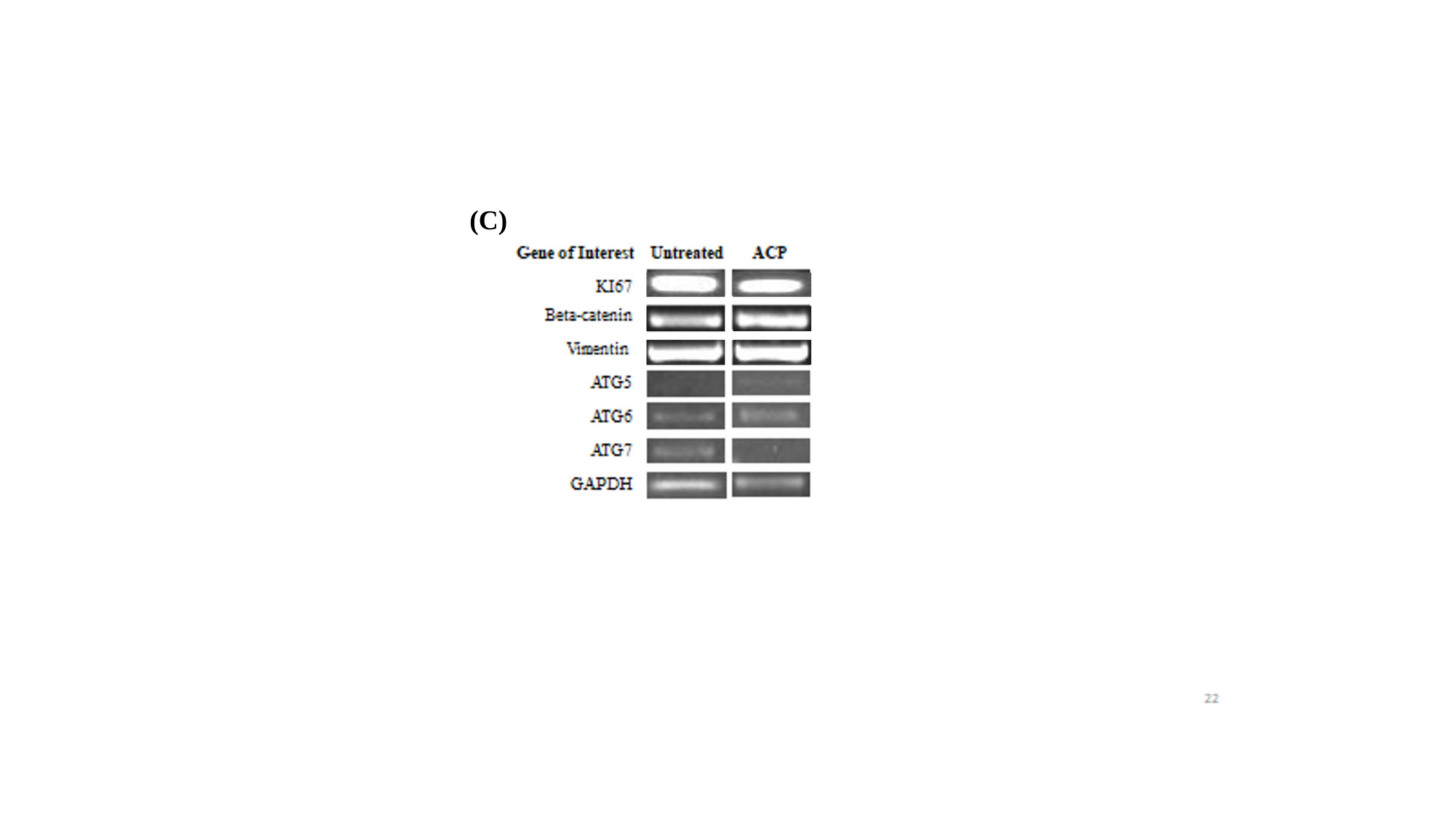

(C)

## Slide 4
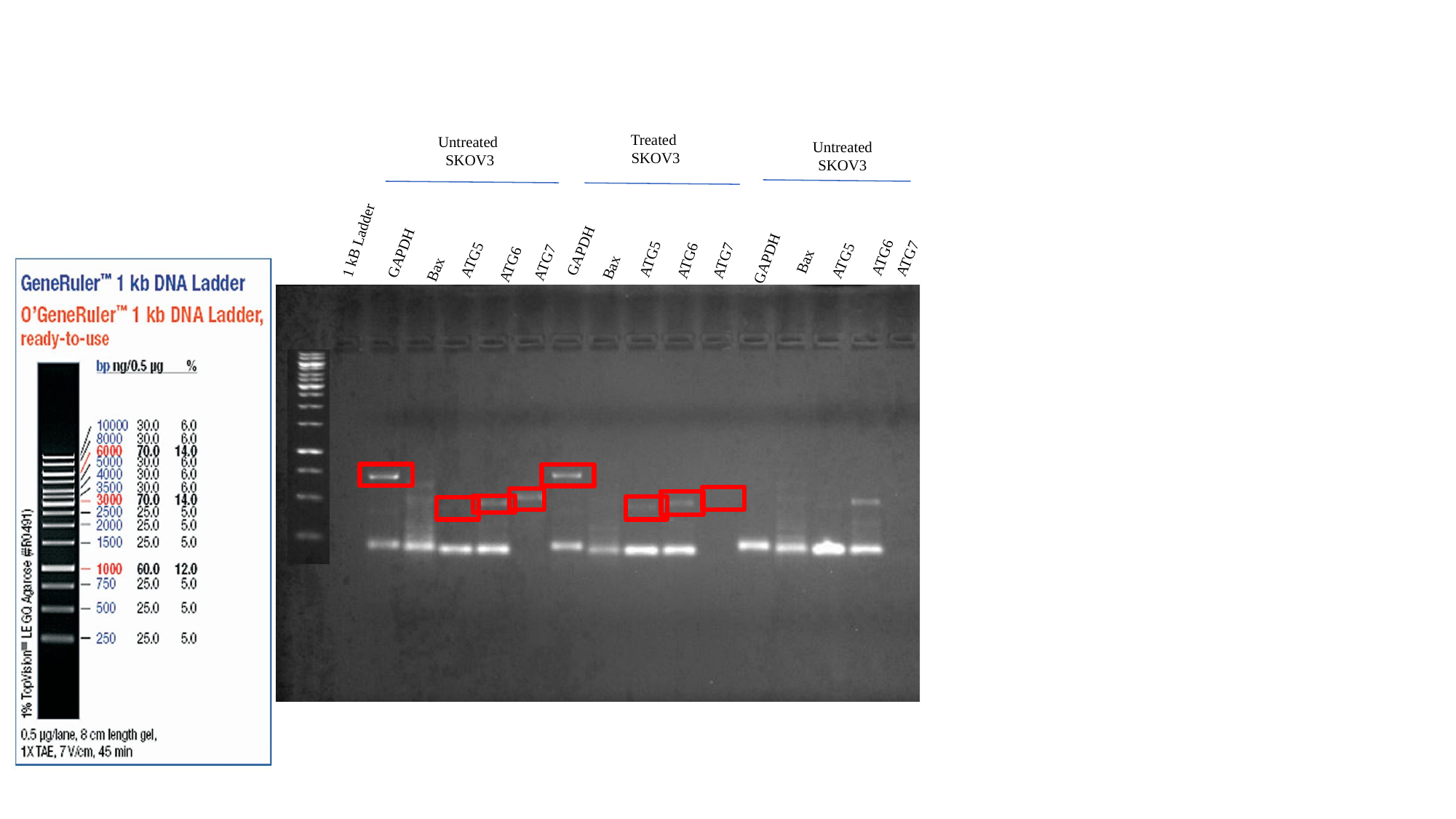

ATG7
ATG5
Treated
SKOV3
Untreated
SKOV3
Untreated
SKOV3
 Bax
 ATG6
 ATG6
 ATG7
 ATG5
Bax
GAPDH
ATG5
 GAPDH
 Bax
 GAPDH
 ATG7
 ATG6
1 kB Ladder

## Slide 5
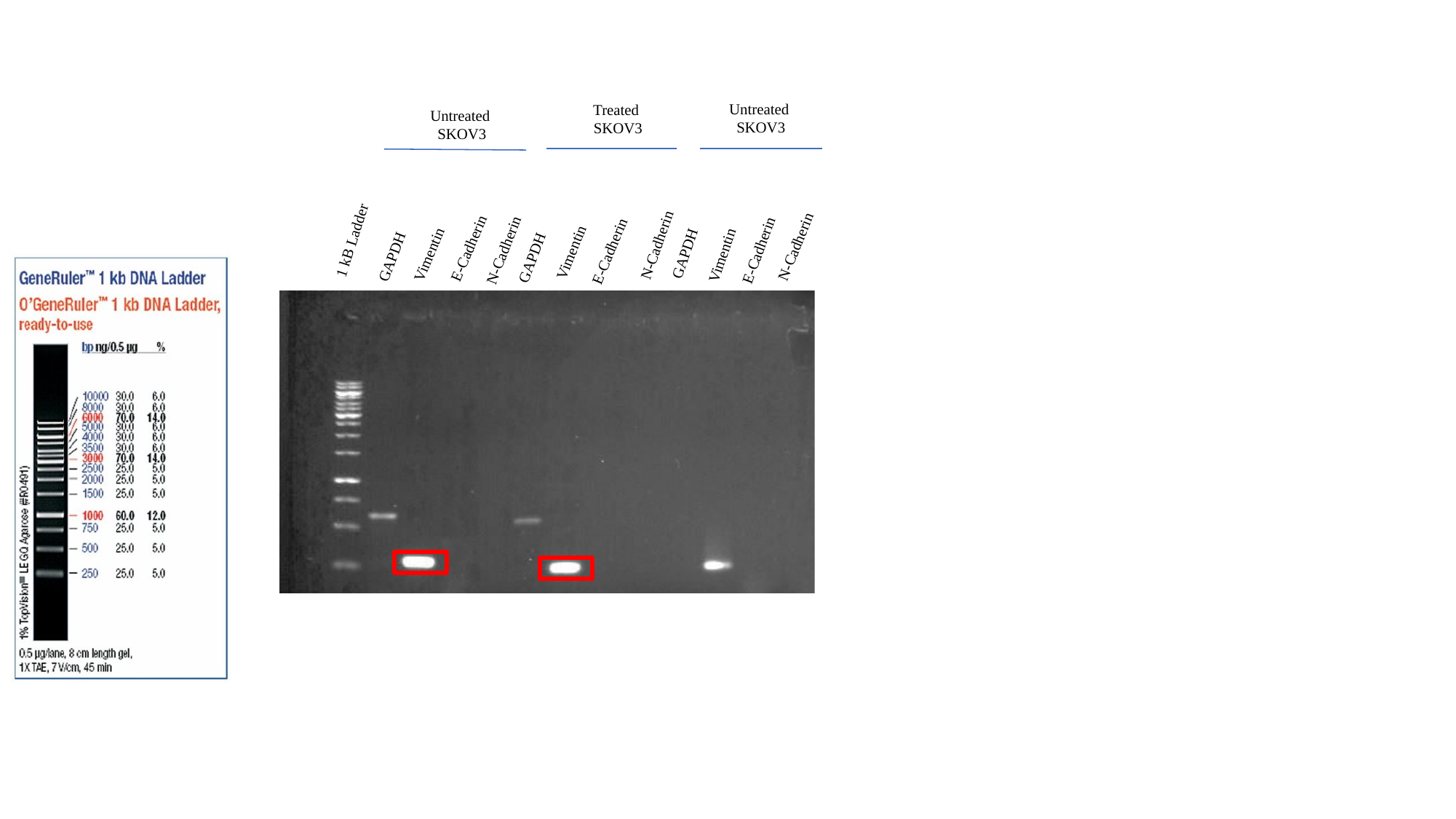

Untreated
SKOV3
Treated
SKOV3
Untreated
SKOV3
 N-Cadherin
 N-Cadherin
N-Cadherin
Vimentin
E-Cadherin
E-Cadherin
E-Cadherin
 Vimentin
 Vimentin
 GAPDH
GAPDH
GAPDH
1 kB Ladder

## Slide 6
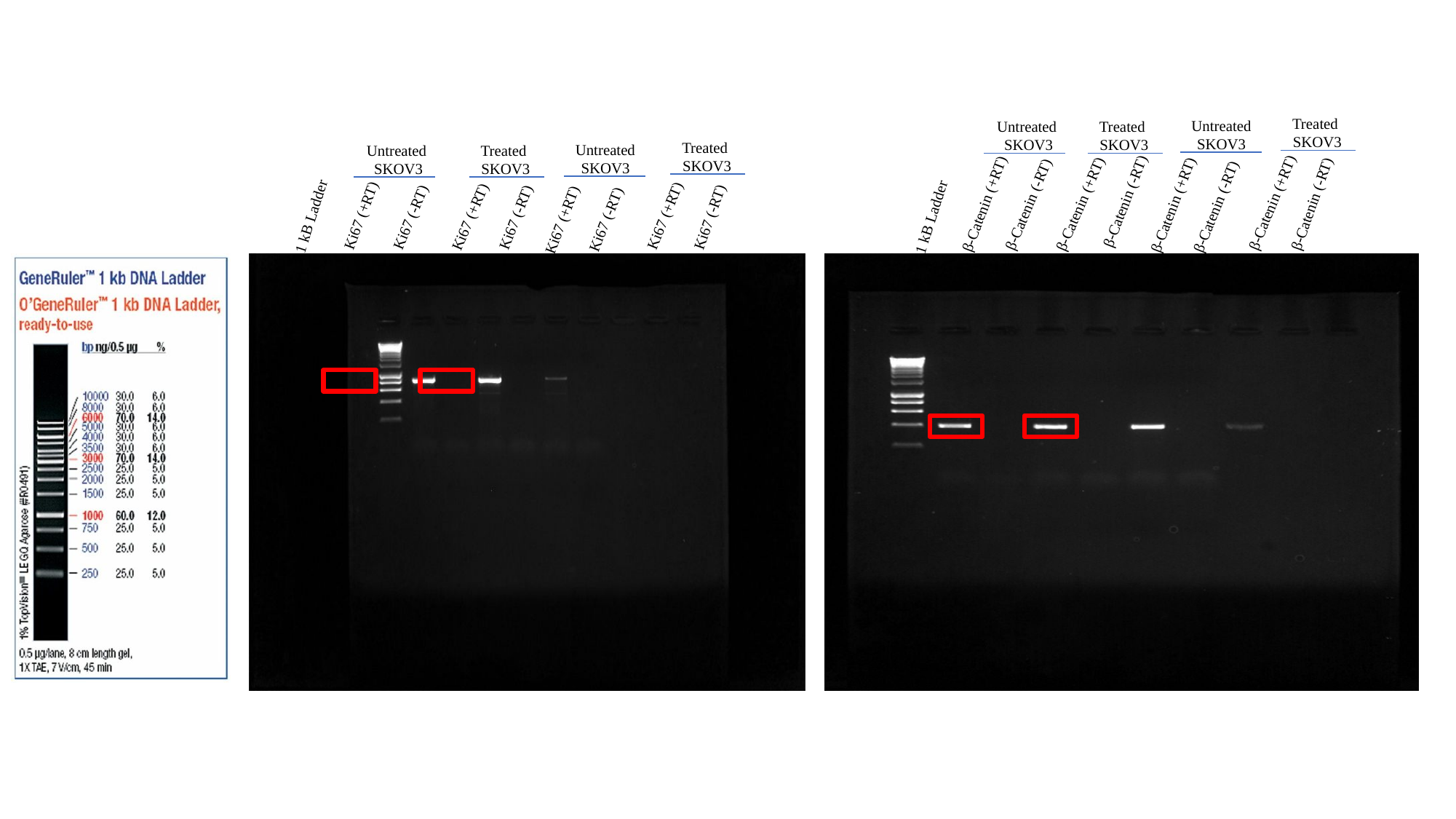

Ki67 (-RT)
β-Catenin (-RT)
β-Catenin (-RT)
Ki67 (-RT)
Treated
SKOV3
Untreated
SKOV3
Untreated
SKOV3
Treated
SKOV3
Ki67 (+RT)
β-Catenin (+RT)
Ki67 (+RT)
β-Catenin (+RT)
Ki67 (-RT)
β-Catenin (-RT)
Ki67 (-RT)
β-Catenin (-RT)
Ki67 (+RT)
Treated
SKOV3
β-Catenin (+RT)
Untreated
SKOV3
Untreated
SKOV3
Treated
SKOV3
β-Catenin (+RT)
Ki67 (+RT)
1 kB Ladder
1 kB Ladder
