# Supplementary material for: Characterization of a novel peptide mined from the Red Sea brine pools and modified to enhance its anticancer activity
Source: BMC Cancer. 2023 Jul 26;23:699. doi: 10.1186/s12885-023-11045-4 (PMC10369728; doi:10.1186/s12885-023-11045-4)
Supplement: Supplementary file 4 — Additional file 4: Table S1. RT-PCR primer parameters: sequences, annealing temperature, cycle number, and amplicon size. [file 12885_2023_11045_MOESM4_ESM.pptx]

## Slide 1
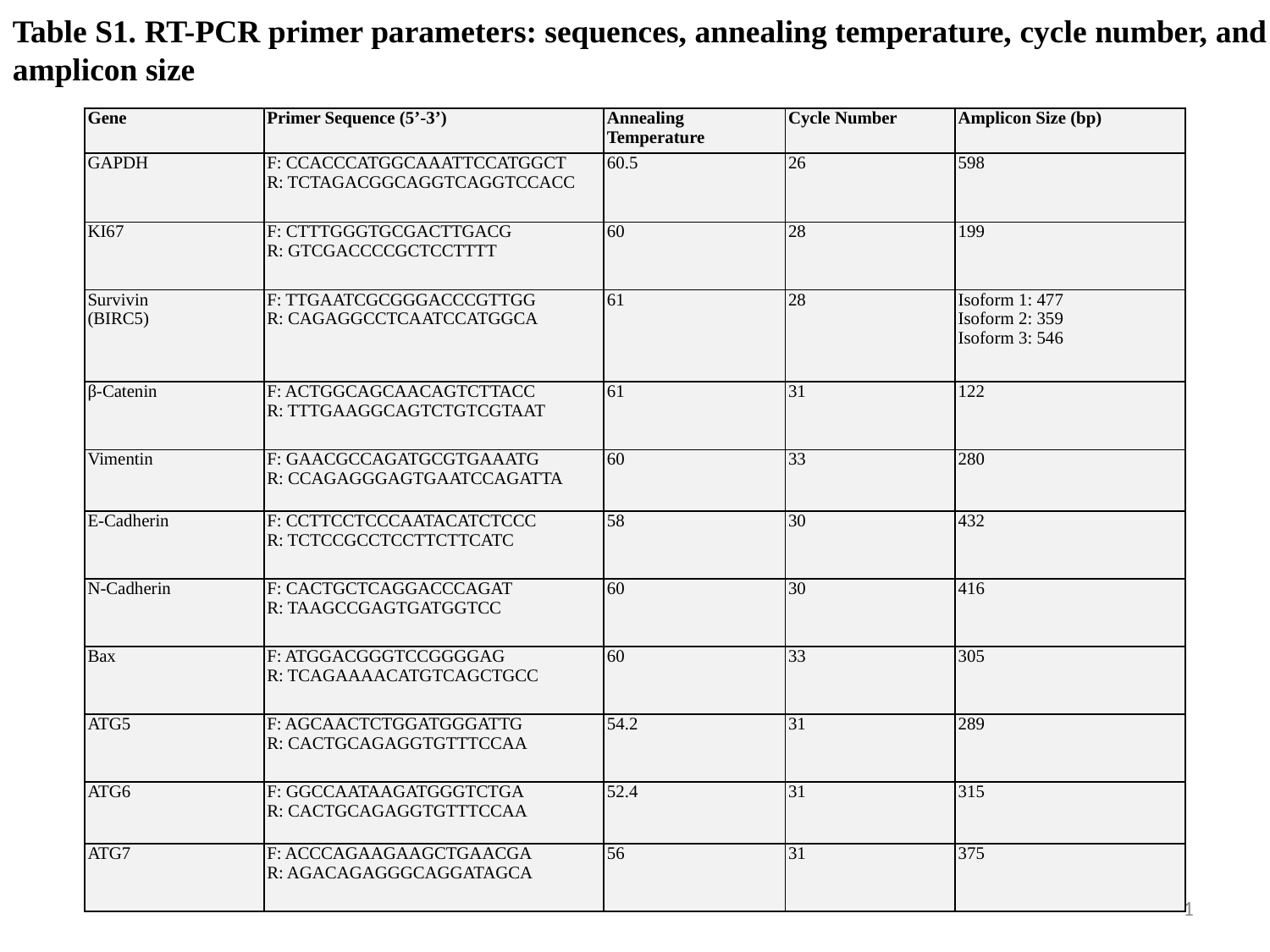

Table S1. RT-PCR primer parameters: sequences, annealing temperature, cycle number, and amplicon size
| Gene | Primer Sequence (5’-3’) | Annealing Temperature | Cycle Number | Amplicon Size (bp) |
| --- | --- | --- | --- | --- |
| GAPDH | F: CCACCCATGGCAAATTCCATGGCT R: TCTAGACGGCAGGTCAGGTCCACC | 60.5 | 26 | 598 |
| KI67 | F: CTTTGGGTGCGACTTGACG R: GTCGACCCCGCTCCTTTT | 60 | 28 | 199 |
| Survivin (BIRC5) | F: TTGAATCGCGGGACCCGTTGG R: CAGAGGCCTCAATCCATGGCA | 61 | 28 | Isoform 1: 477 Isoform 2: 359 Isoform 3: 546 |
| β-Catenin | F: ACTGGCAGCAACAGTCTTACC R: TTTGAAGGCAGTCTGTCGTAAT | 61 | 31 | 122 |
| Vimentin | F: GAACGCCAGATGCGTGAAATG R: CCAGAGGGAGTGAATCCAGATTA | 60 | 33 | 280 |
| E-Cadherin | F: CCTTCCTCCCAATACATCTCCC R: TCTCCGCCTCCTTCTTCATC | 58 | 30 | 432 |
| N-Cadherin | F: CACTGCTCAGGACCCAGAT R: TAAGCCGAGTGATGGTCC | 60 | 30 | 416 |
| Bax | F: ATGGACGGGTCCGGGGAG R: TCAGAAAACATGTCAGCTGCC | 60 | 33 | 305 |
| ATG5 | F: AGCAACTCTGGATGGGATTG R: CACTGCAGAGGTGTTTCCAA | 54.2 | 31 | 289 |
| ATG6 | F: GGCCAATAAGATGGGTCTGA R: CACTGCAGAGGTGTTTCCAA | 52.4 | 31 | 315 |
| ATG7 | F: ACCCAGAAGAAGCTGAACGA R: AGACAGAGGGCAGGATAGCA | 56 | 31 | 375 |
1
