# Supplementary material for: Characterization of a novel peptide mined from the Red Sea brine pools and modified to enhance its anticancer activity
Source: BMC Cancer. 2023 Jul 26;23:699. doi: 10.1186/s12885-023-11045-4 (PMC10369728; doi:10.1186/s12885-023-11045-4)
Supplement: Supplementary file 5 — Additional file 5: Table S2. Summary of IC50, hill coefficient, and R2 values obtained for peptide treatment of HeLa and MCF7 cell lines. [file 12885_2023_11045_MOESM5_ESM.pptx]

## Slide 1
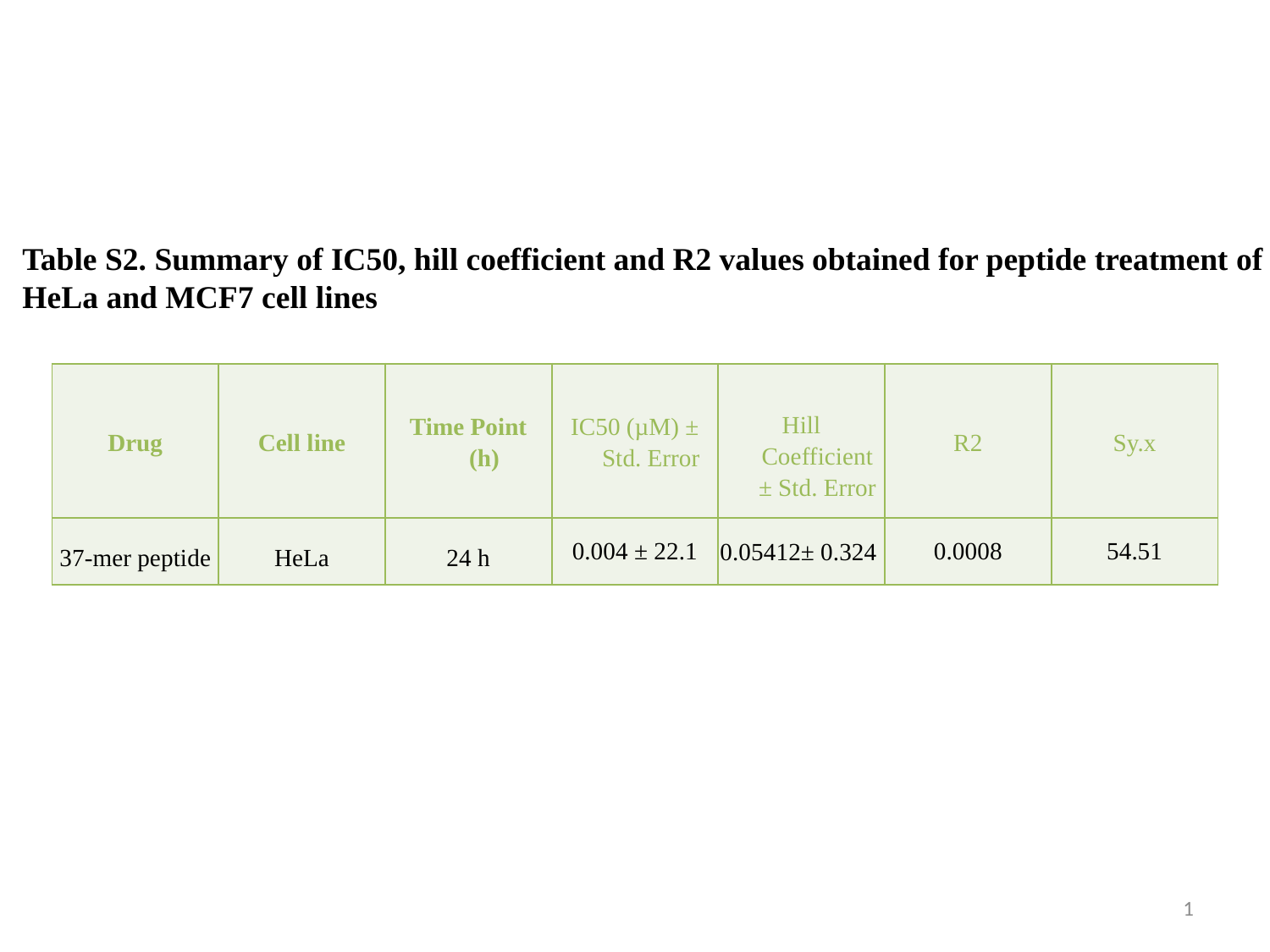

Table S2. Summary of IC50, hill coefficient and R2 values obtained for peptide treatment of HeLa and MCF7 cell lines
| Drug | Cell line | Time Point (h) | IC50 (µM) ± Std. Error | Hill Coefficient± Std. Error | R2 | Sy.x |
| --- | --- | --- | --- | --- | --- | --- |
| 37-mer peptide | HeLa | 24 h | 0.004 ± 22.1 | 0.05412± 0.324 | 0.0008 | 54.51 |
1
