# Supplementary material for: Characterization of a novel peptide mined from the Red Sea brine pools and modified to enhance its anticancer activity
Source: BMC Cancer. 2023 Jul 26;23:699. doi: 10.1186/s12885-023-11045-4 (PMC10369728; doi:10.1186/s12885-023-11045-4)
Supplement: Supplementary file 6 — Additional file 6: Table S3. List of sediments used for Biosamples collection with QC values calculated post quality control for raw sequenced reads. Data available in NCBI SRA under PRJNA299097 and PRJNA193416. [file 12885_2023_11045_MOESM6_ESM.pptx]

## Slide 1
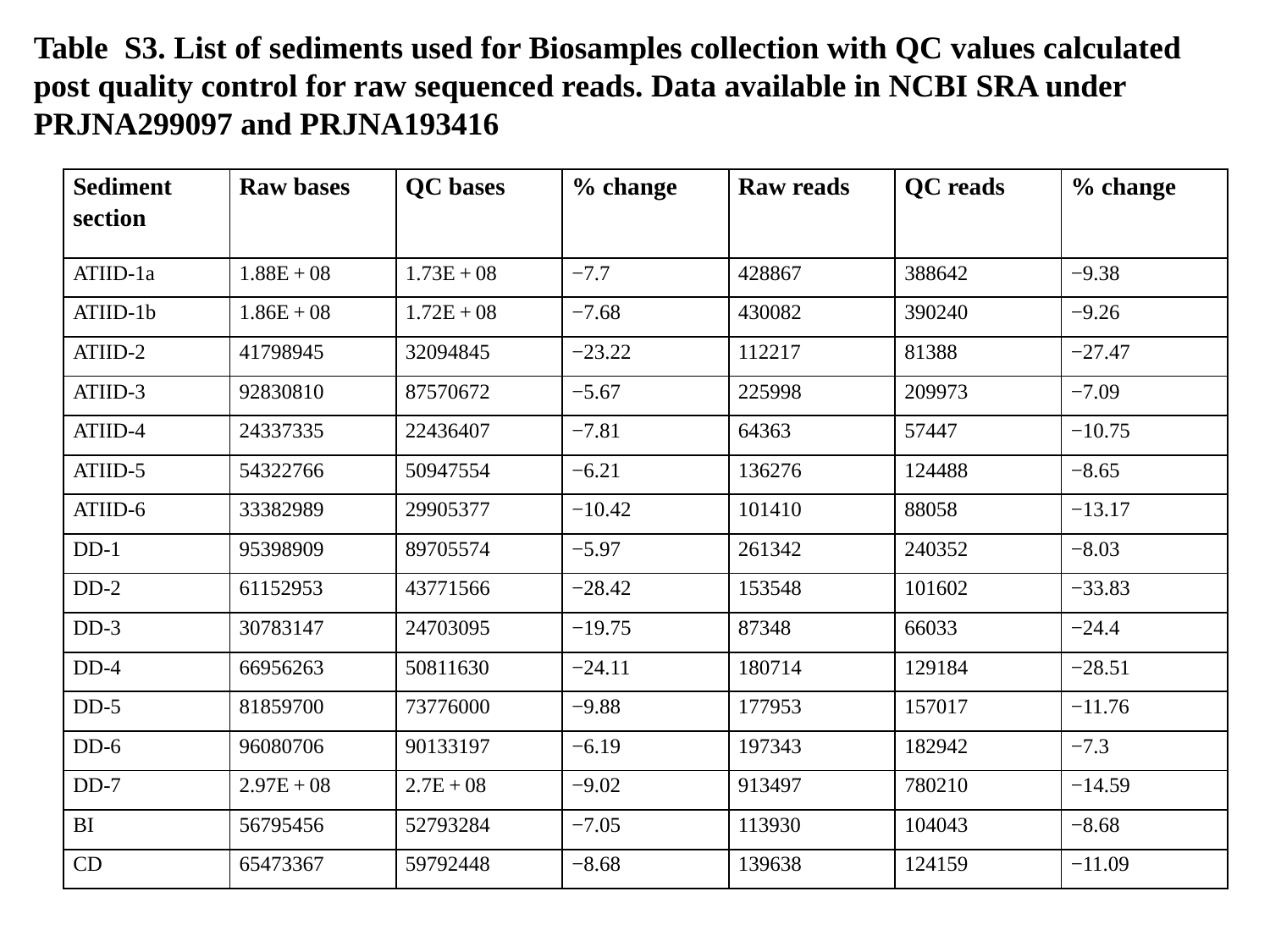

Table  S3. List of sediments used for Biosamples collection with QC values calculated post quality control for raw sequenced reads. Data available in NCBI SRA under PRJNA299097 and PRJNA193416
| Sediment section | Raw bases | QC bases | % change | Raw reads | QC reads | % change |
| --- | --- | --- | --- | --- | --- | --- |
| ATIID-1a | 1.88E + 08 | 1.73E + 08 | −7.7 | 428867 | 388642 | −9.38 |
| ATIID-1b | 1.86E + 08 | 1.72E + 08 | −7.68 | 430082 | 390240 | −9.26 |
| ATIID-2 | 41798945 | 32094845 | −23.22 | 112217 | 81388 | −27.47 |
| ATIID-3 | 92830810 | 87570672 | −5.67 | 225998 | 209973 | −7.09 |
| ATIID-4 | 24337335 | 22436407 | −7.81 | 64363 | 57447 | −10.75 |
| ATIID-5 | 54322766 | 50947554 | −6.21 | 136276 | 124488 | −8.65 |
| ATIID-6 | 33382989 | 29905377 | −10.42 | 101410 | 88058 | −13.17 |
| DD-1 | 95398909 | 89705574 | −5.97 | 261342 | 240352 | −8.03 |
| DD-2 | 61152953 | 43771566 | −28.42 | 153548 | 101602 | −33.83 |
| DD-3 | 30783147 | 24703095 | −19.75 | 87348 | 66033 | −24.4 |
| DD-4 | 66956263 | 50811630 | −24.11 | 180714 | 129184 | −28.51 |
| DD-5 | 81859700 | 73776000 | −9.88 | 177953 | 157017 | −11.76 |
| DD-6 | 96080706 | 90133197 | −6.19 | 197343 | 182942 | −7.3 |
| DD-7 | 2.97E + 08 | 2.7E + 08 | −9.02 | 913497 | 780210 | −14.59 |
| BI | 56795456 | 52793284 | −7.05 | 113930 | 104043 | −8.68 |
| CD | 65473367 | 59792448 | −8.68 | 139638 | 124159 | −11.09 |
